# Hemodynamic forces in the left and right ventricles of the human heart using 4D flow magnetic resonance imaging: Phantom validation, reproducibility, sensitivity to respiratory gating and free analysis software

**DOI:** 10.1371/journal.pone.0195597

**Published:** 2018-04-05

**Authors:** Johannes Töger, Per M. Arvidsson, Jelena Bock, Mikael Kanski, Gianni Pedrizzetti, Marcus Carlsson, Håkan Arheden, Einar Heiberg

**Affiliations:** 1 Lund University, Skane University Hospital, Department of Clinical Sciences Lund, Clinical Physiology, Lund, Sweden; 2 Department of Engineering and Architecture, University of Trieste, Trieste, Italy; 3 Department of Biomedical Engineering, Faculty of Engineering, Lund University, Lund, Sweden; Medical College of Georgia, Augusta, UNITED STATES

## Abstract

**Purpose:**

To investigate the accuracy, reproducibility and sensitivity to respiratory gating, field strength and ventricle segmentation of hemodynamic force quantification in the left and right ventricles of the heart (LV and RV) using 4D-flow magnetic resonance imaging (MRI), and to provide free hemodynamic force analysis software.

**Materials and methods:**

A pulsatile flow phantom was imaged using 4D flow MRI and laser-based particle image velocimetry (PIV). Cardiac 4D flow MRI was performed in healthy volunteers at 1.5T (n = 23). Reproducibility was investigated using MR scanners from two different vendors on the same day (n = 8). Subsets of volunteers were also imaged without respiratory gating (n = 17), at 3T on the same day (n = 6), and 1–12 days later on the same scanner (n = 9, median 6 days). Agreement was measured using the intraclass correlation coefficient (ICC).

**Results:**

Phantom validation showed good accuracy for both scanners (Scanner 1: bias -14±9%, y = 0.82x+0.08, R^2^ = 0.96, Scanner 2: bias -12±8%, y = 0.99x-0.08, R^2^ = 1.00). Force reproducibility was strong in the LV (0.09±0.07 vs 0.09±0.07 N, bias 0.00±0.04 N, ICC = 0.87) and RV (0.09±0.06 vs 0.09±0.05 N, bias 0.00±0.03, ICC = 0.83). Strong to very strong agreement was found for scans with and without respiratory gating (LV/RV: ICC = 0.94/0.95), scans on different days (ICC = 0.92/0.87), and 1.5T and 3T scans (ICC = 0.93/0.94).

**Conclusion:**

Software for quantification of hemodynamic forces in 4D-flow MRI was developed, and results show high accuracy and strong to very strong reproducibility for both the LV and RV, supporting its use for research and clinical investigations. The software including source code is released freely for research.

## Introduction

Blood flow in the human heart is closely linked to the function of valves, great vessels and the myocardium. Therefore, careful analysis of intracardiac blood flow may provide sensitive measures of cardiac function in health and disease. Evaluation of hemodynamic forces using 4D flow MRI and the Navier-Stokes equations was recently introduced to compute the force exchanged between blood and myocardium in the left and right ventricles (LV and RV) [1–3]. Hemodynamic force evaluation was first performed using echocardiography by Pedrizzetti et al. [1], showing that restoration of predominantly longitudinal hemodynamic forces in cardiac resynchronization therapy (CRT) is beneficial for cardiac function and remodeling, and a recent study showed an association between the degree of dyssynchrony and the transversetransverse/longitudinal hemodynamic force ratio measured using 4D flow MRI [4]. Furthermore, intracardiac pressure variations, which are closely related to hemodynamic forces, have been shown to be related to diastolic function and are affected by myocardial ischemia [5,6], suggesting that hemodynamic forces can be a sensitive marker of LV and RV function. By using 4D flow MRI, hemodynamic forces can be quantified noninvasively and in three dimensions, which may provide unique insights into cardiac physiology and pathophysiology. Specifically, the ratio of transverse and longitudinal (basal-apical) and forces has been proposed as a new measure of cardiac health [2–4].

Understanding the accuracy and reproducibility of 4D flow hemodynamic force measurements and its sensitivity to measurement parameters is important for study design and interpretation of results. However, accuracy, reproducibility and sensitivity of hemodynamic force measurements to measurement and analysis conditions have not previously been studied.

Therefore, the aims of this study are to 1) develop software for analysis of hemodynamic forces in 4D flow MRI data, 2) to validate hemodynamic force measurements in a pulsatile flow phantom setup 3) investigate the reproducibility of hemodynamic forces and force ratios measured using 4D flow MRI in the LV and RV, 4) investigate the sensitivity of hemodynamic forces and force ratios to respiratory gating in the 4D flow acquisition, 5) investigate the sensitivity of hemodynamic forces and force ratios to field strength and ventricle delineation method. The developed software for hemodynamic force quantification is provided for free research use, including source code.

## Materials and methods

### Phantom validation

**Fig 1:**
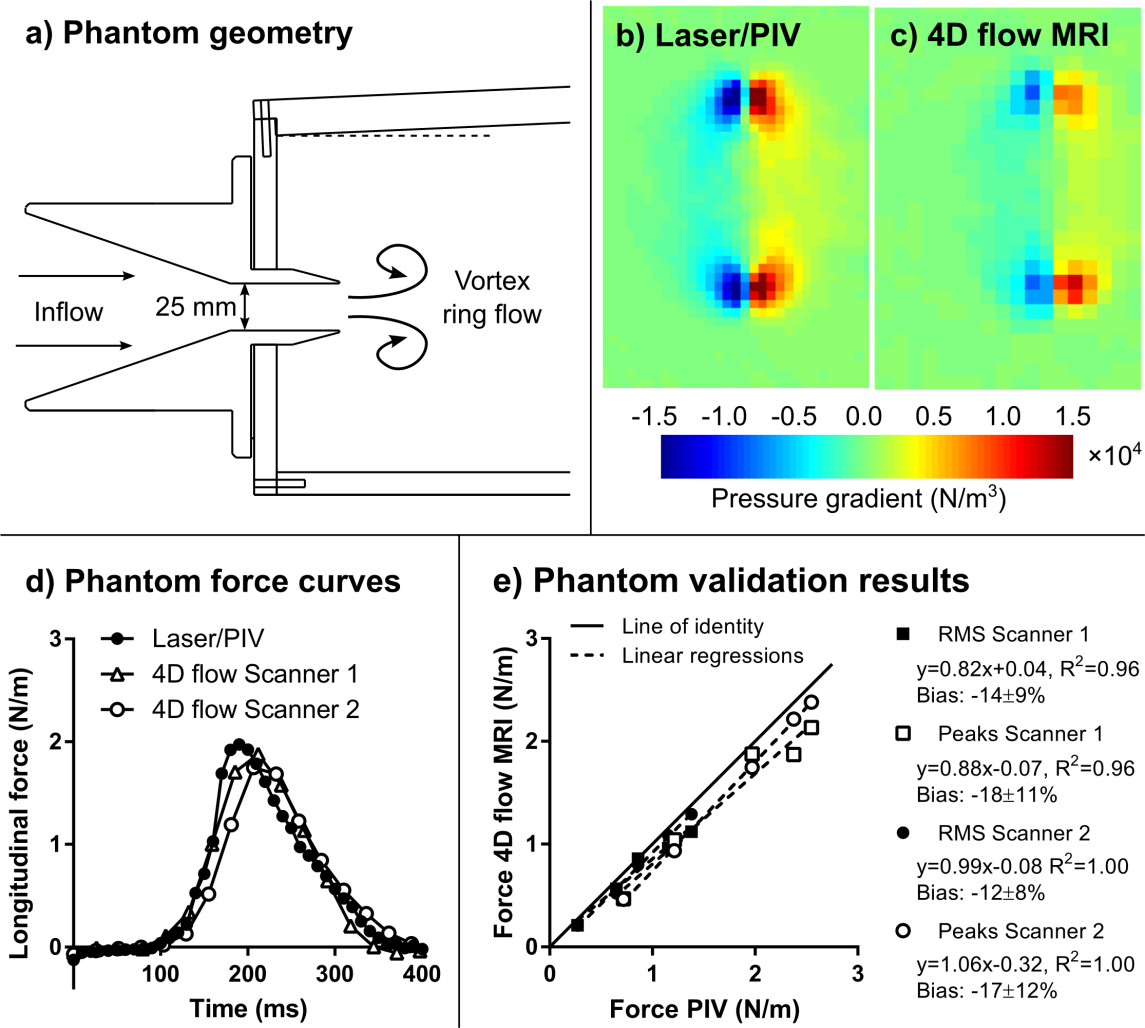
Phantom validation of hemodynamic forces. A pulsatile flow phantom [7] (Panel a) was imaged with a laser-based technique (PIV, particle image velocimetry) and 4D flow MRI. Panels b) and c) show the pressure gradient at one time instant computed from PIV and 4D flow velocities, respectively. Panel d) shows hemodynamic force curves for one pump setting (out of five). Panel e) shows a summary of results for RMS and peak forces on the both scanners, with a slight underestimation for both scanners.

A flow phantom and pump were used to generate pulsatile flow in a 25 mm circular nozzle (Fig 1A) leading into a transparent water tank [7] filled with regular water. Five different pump programs were used, with peak velocities ranging from 21–36 cm/s (Table 1). A particle image velocimetry (PIV) setup consisting of a Continuum MiniLite 532 nm Nd:YAG laser and a FlowMaster 3S camera (LaVision, Bicester, UK) and associated controller hardware was used to measure a reference flow field in the central symmetry plane of the flow. Neutrally buoyant hollow glass spheres (diameter 10 μm) were added to the water until a density of 10–15 particles per PIV interrogation window was achieved. The in-plane velocity components in the two-dimensional measurement plane were computed using the software DaVis 7.2 (LaVision, Bicester, UK). Velocity computation from raw images was performed using cross-correlation in 32×32-pixel interrogation windows with 50% overlap. The resulting spatial resolution was 1.45×1.45 mm and the temporal resolution was 10 ms. A mean of 10 PIV acquisitions was performed for each timeframe to reduce velocity noise. Full details on acquisition and post-processing are given in a previous publication [7]. Furthermore, the phantom setup was scanned on Siemens Aera and Philips Achieva 1.5T MRI scanners using the 4D flow MRI protocols given below. Hemodynamic forces were computed along the main flow direction using the in-plane PIV velocities, and using the in-plane velocities of the corresponding slice through the 4D flow MRI datasets. The same quantification pipeline (further described below) was used for 4D flow and PIV data. Since the PIV setup did not measure through-plane velocities and velocity gradients, these were set to zero (motivated by the axial symmetry of the vortex ring flow). In the phantom validation, the pressure gradient (N/m^3^) is integrated over a plane (m^2^), resulting in the units N/m.

**Table 1:** Pump settings for phantom experiments.

Pump program	1	2	3	4	5
Pump frequency (beats per minute)	57	57	57	57	57
Pulse volume (ml)	12.3	18.4	24.5	30.7	36.8
Inflow peak velocity (cm/s)	21.0	27.1	31.1	33.0	35.6

### Study population and CMR protocol

Twenty-three (n = 23) healthy volunteers were included. The study was approved by the regional ethical review board in Lund, Sweden, and all subjects provided written informed consent after receiving written and oral information about the study. Subjects were recruited among colleagues, friends and family of the authors during 2009 (n = 6), 2011 (n = 8), and 2016 (n = 9). The total number of approached subjects and the drop-out rate was not recorded. The criterion for inclusion was that the subject should be willing and able to undergo an MRI scan as a healthy volunteer. Exclusion criteria were: history of cardiovascular or systemic disease, claustrophobia, pregnancy, or metal implants or devices that are not considered safe in an MRI environment. A subset of the subjects was included in a previously published study [3]. Subject characteristics are presented in Table 2.

**Table 2:** Subject characteristics.

	Healthy volunteers (n = 23)
Age (years)	30±7 (range 23–52)
Gender (% F)	9F, 14M (39%)
Weight (kg)	73±14
Height (cm)	178±10
BSA (m^2^)	1.89±0.23
Heart rate (bpm)	60±8
EDV (ml)	175±36
EDV/BSA (ml/m^2^)	92±13
ESV (ml)	70±17
SV (ml)	105±22
EF	60±8%

Values are given as mean±standard deviation (SD). F = female, M = male. bpm = beats per minute. BSA = body surface area (Mosteller formula). Heart rate was recorded by the MRI scanner at the 4D flow examination.

All subjects underwent cardiac magnetic resonance examinations (MR) at 1.5T (Philips Achieva, Philips Healthcare, Best, The Netherlands, and/or Magnetom Aera, Siemens Healthcare, Erlangen, Germany) including balanced steady-state free-precession (bSSFP) cine images in long-axis and short-axis views. A subset of subjects was scanned at 3T (Philips Achieva, Philips Healthcare, Best, The Netherlands). Typical imaging parameters for cine scans: slice thickness 8 mm, no slice gap, field of view 340–400 mm, acquired matrix 192x180, reconstructed matrix 256x256 or 384x384, reconstructed in-plane resolution 1.5x1.5 mm^2^, TR/TE 1.4/2.8 ms, flip angle 60°, bandwidth/pixel 1040 or 930 Hz, temporal resolution 30 ms. An experienced observer verified normal anatomy, global function, wall motion and valve function in all subjects.

For subjects imaged on Philips scanners (Scanner 1, n = 14), magnetic resonance 4D flow was performed at rest using a three-dimensional gradient echo sequence [8], based on a 4D flow consensus statement [9] and previously validated in our lab [7,10–13]. Typical parameters were: four-point referenced velocity encoding, spatial resolution 3×3×3 mm^3^, acquired matrix size 80×80, 52 axial slices, flip angle 8°, TR/TE 6.3/3.7 ms, bandwidth/pixel 340 Hz, VENC 100 cm/s, acquired temporal resolution 50 ms, 40 reconstructed time phases, slice oversampling factor 1.4, no partial Fourier acquisition, SENSE = 2 in the anterior-posterior direction and temporal segmentation factor 2. In a further series of subjects scanned on another Philips scanner (n = 9), a modified sequence with SENSE factor of 4 elliptical k-space shutter and bandwidth 723 Hz/pixel was used to enable faster scans for a patient protocol (SENSE factor 2 in the anterior-posterior phase-encoding direction and 2 in the superior-inferior slice-encoding direction). For SENSE = 2 (n = 14), scan times were 47±19 minutes with respiratory gating, and 27±9 minutes without. For SENSE = 4 (n = 9), scan times were 18±3 minutes with respiratory gating and 11±2 minutes without.

For subjects imaged on the Siemens scanner (Scanner 2, n = 9), the 4D flow protocol was based on a prototype sequence, with parameters similar to the Philips protocol: spatial resolution 3×3×3 mm^3^ (acquired and reconstructed), matrix size 80×80, 64 axial slices, flip angle 8°, TR/TE 5.7/3.5 ms, bandwidth 560 Hz/pixel, VENC 100 cm/s, partial Fourier factor 0.75 in phase and slice directions, no slice oversampling, acquired temporal resolution 46 ms, 40 reconstructed time phases, GRAPPA factor 4 (factor 2 in anterior-posterior phase encode direction and 2 in superior-inferior slice encode direction) and temporal segmentation factor 2. Scan times were 10±3 minutes with respiratory gating and 6±1 minutes without.

For 4D flow on both vendors, retrospective ECG triggering was used, i.e. the 4D flow data covers the whole cardiac cycle including the late atrial filling of the ventricles. Respiratory gating was performed using a navigator acquisition at the lung-liver interface. Spatial alignment of cine and 4D flow images was assessed visually and manually adjusted when needed. Phase background correction was performed by subtracting a first-order polynomial fit of velocities in static tissue [14]. Phase aliasing artifacts were unwrapped automatically, or manually when needed.

### Reproducibility and sensitivity

The following investigations were performed to assess the reproducibility and sensitivity of in vivo hemodynamic force quantification to different variables in data acquisition and analysis:

1. *Reproducibility*: In 8 subjects, MRI scans were performed on scanners from both vendors on the same day.
2. *Scan-rescan*: In 9 subjects, MRI scans were performed twice on the same scanner on separate days (range 1–12 days, median 6 days)
3. *Respiratory gating*: In 17 subjects, 4D flow was performed both with and without respiratory gating in the same scan session (i.e. the subject did not leave the scanner).
4. *Field strength*: In 6 subjects, MRI scans were performed at 1.5T and 3T on the same day in random order.
5. *Segmentation*: In 12 subjects, hemodynamic force analysis was performed based on manual and automatic LV segmentations [15].

The investigation of respiratory gating was motivated by a previous study where data both with (Resp+) and without (Resp-) respiratory gating were used interchangeably [3], and the fact that using a Resp- sequence gives significantly shorter scan times [11], giving a potentially more useful method for clinical purposes.

### Definition of cardiac cycle events

The onset of systole (S) was defined using the electrocardiogram (ECG) triggering function of the MR scanner, which triggers image acquisition on the R wave of the ECG signal. The end of systole (and onset of diastole) was determined using 4D flow data as the time phase between cessation of flow in the aortic root and initiation of transmitral flow at the AV-plane level. For comparison of hemodynamic force curves between subjects, all curves were resampled in time to an average heartbeat.

### Freely available analysis software

The software for hemodynamic force analysis used in this study is provided for free use in research as part of the source code distribution of Segment [16] software package (from version 2.0R5439), available for free download according to instructions in Supporting File S1 Appendix. Matlab source code is provided. Software and source code is provided for free use, provided that the present publication is cited for hemodynamic force analysis, and that the relevant publications are cited for relative pressure field calculation [17,18]. Instructions for use and further details are provided in Supporting File S1 Appendix. The analysis software is designed to work on 4D flow data from all vendors, provided that the cine and 4D flow MRI data can be loaded into Segment. At the time of writing, this has been tested for Philips and Siemens 4D flow data.

### Quantification of hemodynamic forces

**Fig 2:**
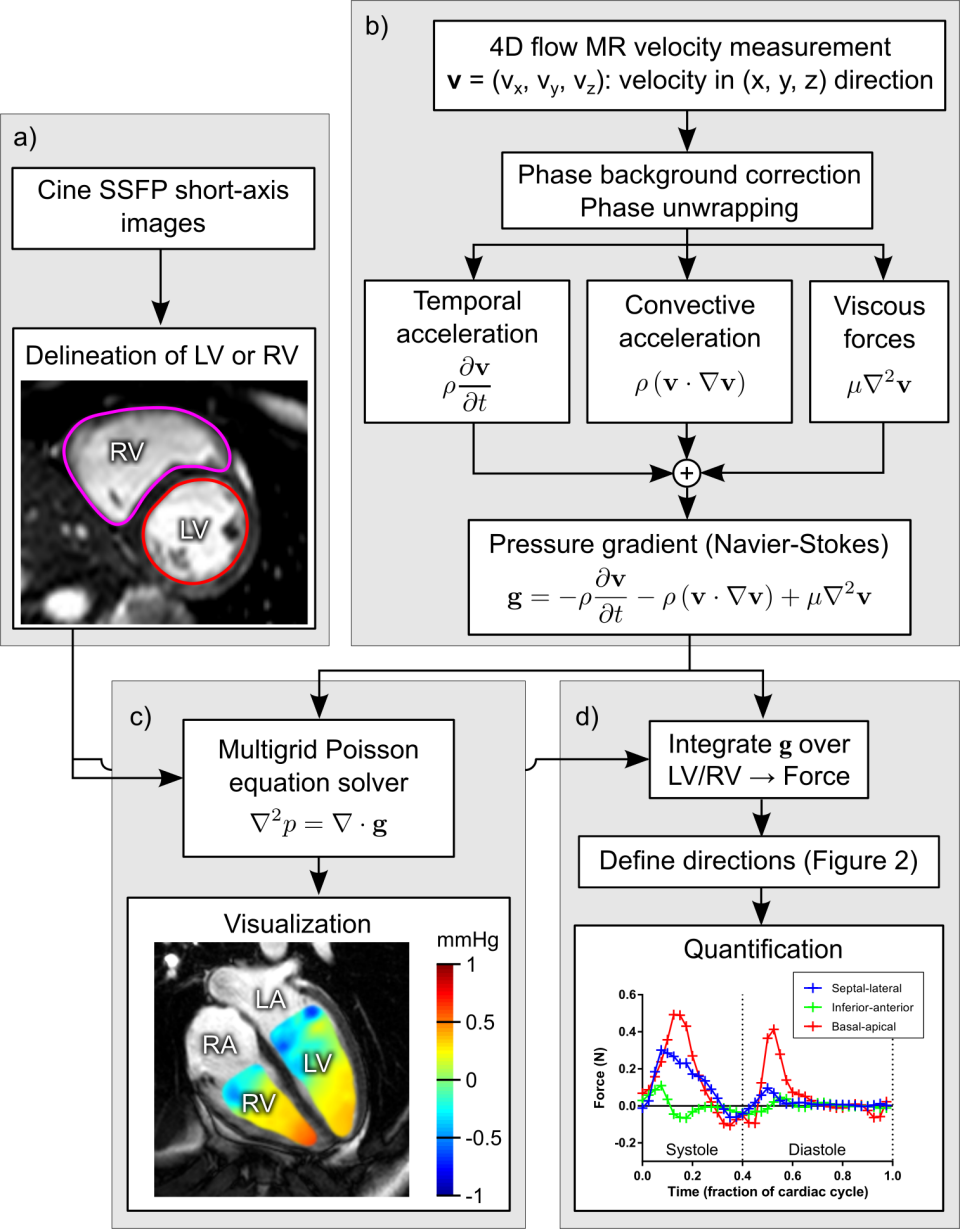
Algorithm for quantification and visualization of hemodynamic forces. Panel a) shows acquisition of cine short-axis SSFP images of the LV and RV. The LV and RV were delineated over the whole cardiac cycle. Panel b) shows acquisition of 4D flow, correction for phase offsets and phase wraps, and subsequent calculation of the pressure gradient (**g**) using the Navier-Stokes equation. Panel c) shows calculation of the intraventricular pressure field using a multigrid Poisson equation solver [17]. Panel d) shows quantification of hemodynamic forces (here shown for LV).

The computation of hemodynamic forces from 4D flow data is shown in Fig 2. First, the LV or RV is delineated over the whole cardiac cycle in short-axis images either manually or using a previously described automatic algorithm [15] (LV only), available in the software package Segment [16]. Manual segmentations were performed by tracing the endocardial boundary in all timeframes while excluding papillary muscles. The epicardial boundary was traced in end-systole and end-diastole to ensure that left ventricular mass was constant as a quality check for the delineations. For the automatic algorithm [15], the required user input is 1) the slices containing LV myocardium, and 2) the center of the LV in the short-axis plane. Thereafter a time-resolved LV segmentation is computed using a deformable model and the expectation-maximization method. Minor manual corrections to the automatic segmentation were applied when needed, predominantly in basal slices of the LV. The pressure gradient **g** was computed using the Navier-Stokes equation as

$$g=-\rho \frac{\partial v}{\partial t}-\rho \left(v∙\nabla v\right)+\mu \nabla^{2}v,$$ (Eq 1)

where **v** is the velocity measured using 4D flow. Blood density was set to ρ = 1.05 g/cm^3^ [19] and viscosity was set to μ = 4×10^−3^ Ns/m^2^. Spatial and temporal derivatives were computed using centered finite differences on the rectilinear grid implied by the 4D flow spatial and temporal resolution. The units of **g** are Newtons per cubic meter (N/m^3^). The hemodynamic force was computed as the integral of **g** over the LV or RV, which results in the unit Newton (N).

**Fig 3:**
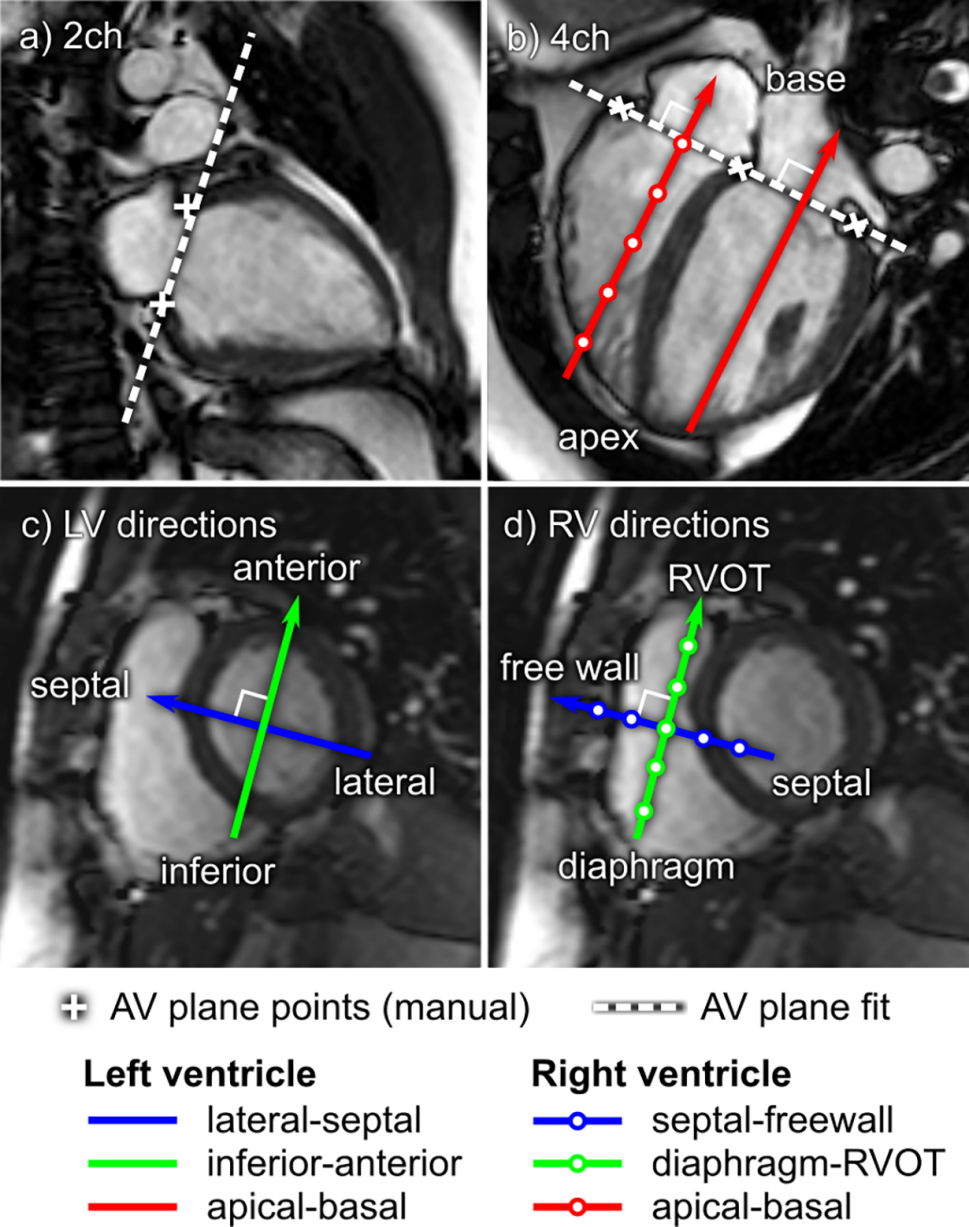
Semi-automated definition of force directions. Panels a) and b) show how atrioventricular plane (AV-plane) points were defined manually in long-axis images (white crosses). A plane was then automatically fit to the points to represent the AV-plane (white dashed line). The apical-basal direction (red) was then defined as orthogonal to the AV-plane (Panel b). Panel c) shows LV transverse directions, and panel d) shows RV transverse directions. Panels c) and d) shows how the lateral-septal and septal-freewall (blue) directions were aligned with the three-chamber slice direction. The inferior-anterior and diaphragm-RVOT (green) directions were then defined as orthogonal to the lateral-septal and septal-freewall directions.

Fig 3 shows how the apical-basal and transverse directions in the LV and RV were determined semi-automatically, following Arvidsson et al. [3]. The basal-apical direction was defined as perpendicular to the atrioventricular plane (AV-plane), computed from AV-plane landmarks placed in long-axis images (Fig 3A and 3B). In the LV, the septal-lateral direction was defined as perpendicular to the basal-apical direction and parallel to the 3-chamber slice location. This alignment of the septal-lateral direction was chosen because it intersects the mitral annulus and left ventricular outflow tract (LVOT), where most of the physiologically meaningful flow occurs. This direction therefore captures most of the important LV flow features. The inferior-anterior direction was defined as perpendicular to both the basal-apical and septal-lateral directions. In the RV, the septal-freewall direction was defined as parallel to the LV lateral-septal direction, and the diaphragm-RVOT as parallel to the LV inferior-anterior direction.

Hemodynamic force peaks were computed as the maximum absolute force in a cardiac phase. The temporal root mean square (RMS) of the hemodynamic force was computed for each direction using the formula

$$\mathrm{RMS}=\sqrt{\frac{1}{N}\overset{N}{\underset{n=1}{\sum }}{\left|f_{n}\right|}^{2}},$$ (Eq 2)

where *N* is the number of time frames, *f*_*n*_ is the force in time frame *n*, and *N* is the number of timeframes in a cardiac phase. The RMS of the hemodynamic force in each direction was computed for systole and diastole separately.

We quantified the relative magnitude of transverse (inferior-anterior and septal-lateral) and longitudinal (basal-apical) forces using the ratio between transverse and longitudinal forces using the two previously published methods [2,3]. First, the ratio was computed based on RMS values [3]:

$$R_{\mathrm{RMS},\mathrm{LV}}=\frac{\sqrt{{\mathrm{RMS}}_{\inf-\mathrm{ant}}^{2}+{\mathrm{RMS}}_{\mathrm{sep}-\mathrm{lat}}^{2}}}{{\mathrm{RMS}}_{\mathrm{base}-\mathrm{apex}}},$$ (Eq 3)

where *RMS*_base-apex_, *RMS*_inf-ant_ and *RMS*_sep-lat_ are the RMS force components in the basal-apical, inferior-anterior and septal-lateral directions, respectively. The ratio was computed for systole and diastole separately and for both the LV and RV. For the RV, the diaphragm-RVOT and septal-freewall directions were used in the numerator:

$$R_{\mathrm{RMS},\mathrm{RV}}=\frac{\sqrt{{\mathrm{RMS}}_{\mathrm{sep}-\mathrm{freewall}}^{2}+{\mathrm{RMS}}_{\mathrm{diaphragm}-\mathrm{RVOT}}^{2}}}{{\mathrm{RMS}}_{\mathrm{base}-\mathrm{apex}}}$$ (Eq 4)

We also computed the LV force ratio based on peak values [2]:

$$R_{\mathrm{peaks},\mathrm{LV}}=\frac{\max\left|f_{\mathrm{sep}-\mathrm{lat}}\right|}{\max\left|f_{\mathrm{base}-\mathrm{apex}}\right|}$$ (Eq 5)

where *f*_*base-apex*_ and *f*_*sep-lat*_ are the forces in basal-apical and septal-lateral directions, respectively. The inferior-anterior was not included in the peak ratio method in a previous study, presumably motivated by its small magnitude in healthy controls [2]. In the RV, the main transverse force component is along the diaphragm-RVOT direction, and therefore the RV peak force ratio was computed using the maximum diaphragm-RVOT force in the numerator for the peak force method:

$$R_{peaks,RV}=\frac{max|f_{diaphraghm-RVOT}|}{max|f_{base-apex}|}$$ (Eq 6)

### Pressure field calculation

**Fig 4:**
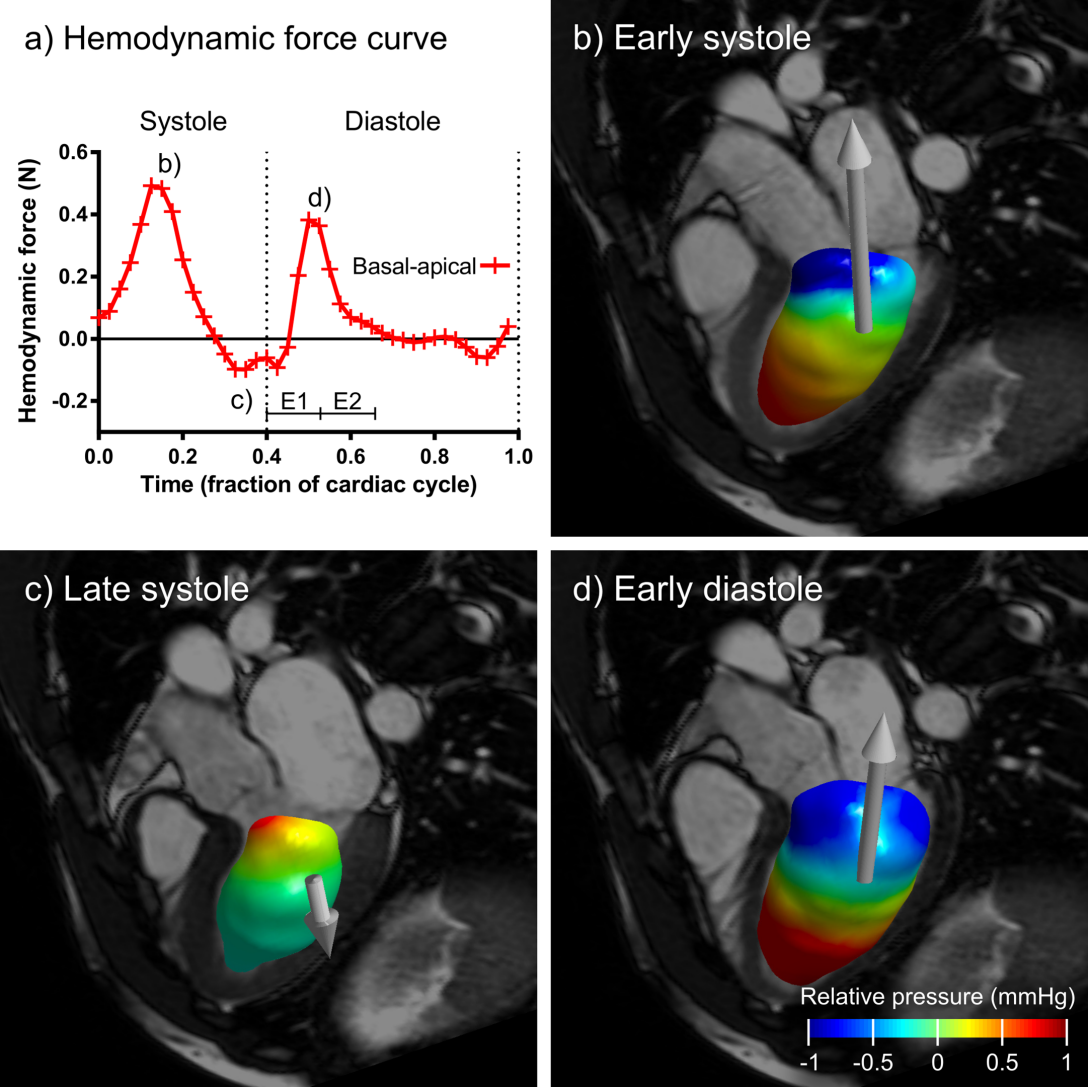
Visualization of LV hemodynamic forces and relative pressure fields in a healthy volunteer. Panel a) shows the basal-apical component of the hemodynamic force and panels b), c), and d) show the relative pressure field and the hemodynamic force vector (white arrow) in three timeframes. The positive basal-apical force component during early systole corresponds to the high apical pressure and low basal pressure in Fig 4B and similarly for Fig 4D. In Fig 4C, the negative basal-apical force is reflected in the low apical pressure and high basal pressure. An animated version is available in Supporting File S1 Movie. *E1 = first half of early rapid filling of LV*, *time fraction 0*.*393–0*.*529*, *E2 = second half of early rapid filling of LV*, *time fraction 0*.*529–0*.*664*.

Pressure field calculation was performed for visualization purposes (Figs 2 and 4). The pressure Poisson equation (PPE) was solved using a previously published multigrid solver [17,18] with source code freely available online (*antigradient2*.*c*, available at https://github.com/GunnarFarneback/spatial_domain_toolbox).

### Statistical methods

Systematic differences were tested using the paired non-parametric Wilcoxon matched-pair signed rank test. Agreement was quantified using the intra-class coefficient (ICC). The ICC is a summary measure of agreement, where 1 signifies perfect agreement and 0 complete lack of agreement. Following a previously published review [20], the level of agreement is considered *poor* for ICC between 0.00 and 0.30, *weak* between 0.31 and 0.50, *moderate* between 0.51 and 0.70, *strong* between 0.71 and 0.90 and *very strong* between 0.91 and 1.00. The same ranges were used for R^2^ values in linear regressions.

The ICC was computed using a linear random-effects model as follows [21,22]. For the measurements *m*_*ij*_ of *n* quantities using two methods (*i = 1*..*n*, *j = 1*,*2)*, we have the following model:

$$m_{ij}=A+M_{j}+p_{i}+\varepsilon_{ij}$$ (Eq 7)

where *A* is the global average, and variance components due to method *M* (e.g. *M*_*1*_ is 1.5T and *M*_*2*_ is 3T data, or equivalent for the other comparisons), data point *p*, and residual/error *ε* are all treated as random effects. Variance components were estimated using a maximum-likelihood algorithm in the package *lme4* in the statistical software package R [23,24]. The ICC was then computed as the following ratio of estimated variances:

$$ICC=\frac{{\tilde{\sigma }}_{p}^{2}}{{\tilde{\sigma }}_{p}^{2}+{\tilde{\sigma }}_{M}^{2}+{\tilde{\sigma }}_{\varepsilon }^{2}}$$ (Eq 8)

Agreement was also analyzed using linear regression and Bland-Altman analysis, including mean and SD of differences and mean and SD of differences in percent.

According to recent standardization of terminology by the Quantitative Imaging Biomarkers Alliance (QIBA) [25,26], *repeatability* is defined as the measurement precision using the *same* measurement procedure, operators, measuring system, operating conditions and the physical location, and measurements on the same subjects over a short period of time. In contrast, *reproducibility* is defined as measurement precision using *different* locations, operators and measuring systems on the same subjects, over a short period of time. In this study, the scans of the same subjects on two different scanners on the same day fits best to the term reproducibility, which is why the term reproducibility was used. Since none of the comparisons in this study fit well to the criteria for repeatability, this term was avoided.

## Results

### Phantom validation

Fig 1 shows results from the phantom validation, including pressure gradient fields from PIV and 4D flow (Fig 1B and 1C), force time curves for PIV and 4D flow MRI (Fig 1D) and comparison of PIV and 4D flow MRI quantitative parameters (Fig 1E). Good agreement was found, with a small underestimation of forces (Scanner 1 RMS: –14±9%, Scanner 2 RMS: -12±8%).

### In vivo hemodynamic forces

**Fig 5:**
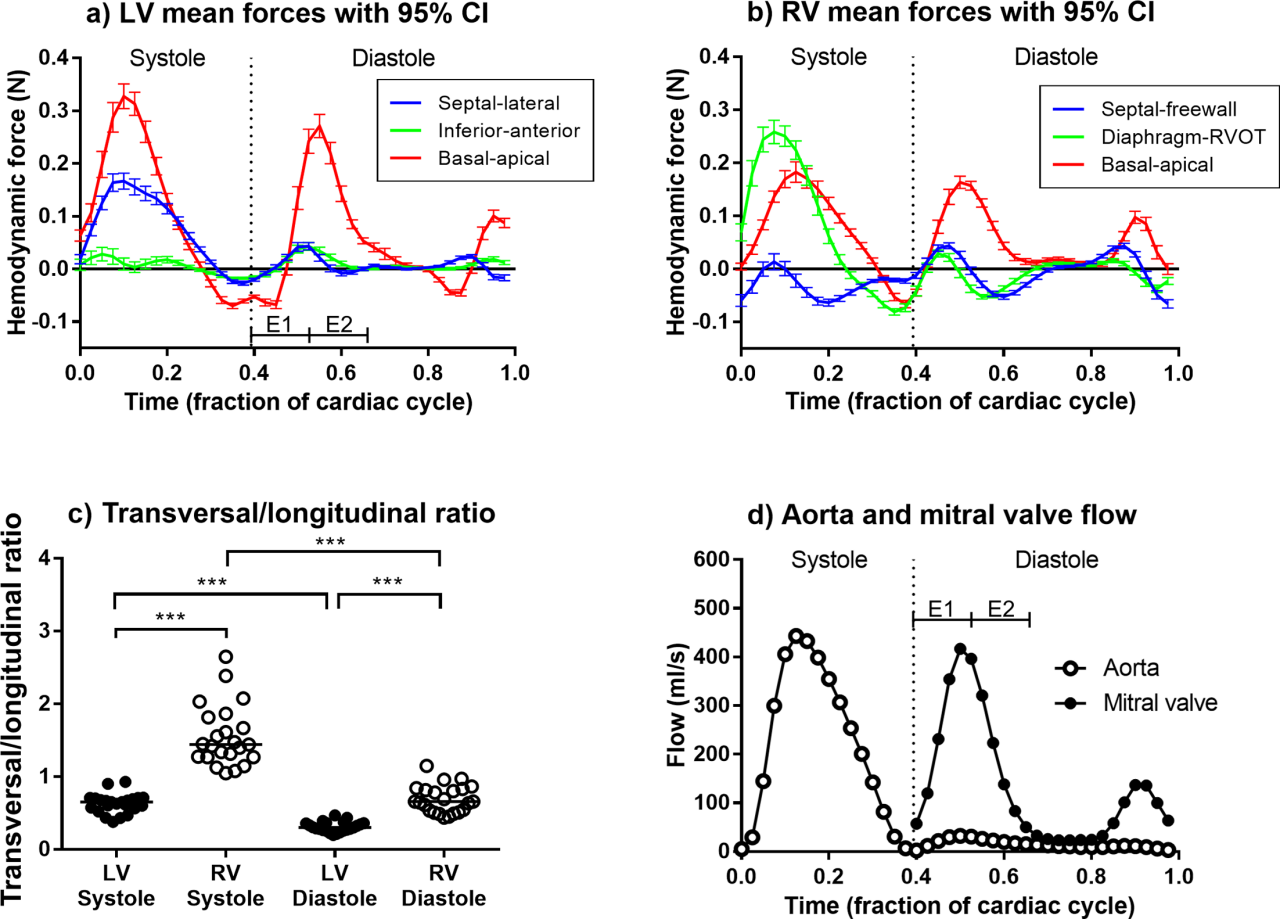
Hemodynamic force curves from all subjects. Panel a) shows mean LV force curves for all three directions, including 95% confidence intervals (CI) for the mean force at each point in the cardiac cycle. Panel b) shows mean RV force curves for all three directions. Panel c) shows the ratio between longitudinal and transverse forces in systole and diastole in the LV and RV (based on RMS forces). Panel d) shows mean transmitral and aortic flow in all subjects as a reference for timing of hemodynamic forces with respect to intracardiac flow. *E1 = first half of early rapid filling of LV*, *time fraction 0*.*393–0*.*529*, *E2 = second half of early rapid filling of LV*, *time fraction 0*.*529–0*.*664*.

Fig 4 shows a visualization of LV hemodynamic forces and intraventricular pressure fields in one selected subject. The first and second half of the early rapid filling phase of the LV is indicated by the labels E1 (time fraction 0.393–0.529) and E2 (0.529–0.664). Table 3 shows mean hemodynamic forces and force ratios in all subjects. The full dataset of force curves derived from 4D flow images is available in Supporting File S2 Appendix. Fig 5 shows LV and RV hemodynamic forces separated into septal-lateral, inferior-anterior and basal-apical components over the whole cardiac cycle for all subjects, average force curves in all three directions and the transverse/longitudinal ratio in systole and diastole.

**Table 3:** Hemodynamic forces and transverse/longitudinal force ratio in the LV and RV.

	RMS systole	RMS diastole	Peaks systole	Peaks diastole
LV hemodynamic forces				
Septal-lateral (N)	0.11±0.04	0.03±0.01	0.19±0.07	0.07±0.03
Inferior-anterior (N)	0.04±0.02	0.02±0.01	0.09±0.05	0.06±0.03
Basal-apical (N)	0.18±0.06	0.12±0.03	0.35±0.11	0.31±0.08
RV hemodynamic forces				
Septal-freewall (N)	0.06±0.02	0.04±0.01	0.11±0.05	0.09±0.03
Diaphragm-RVOT (N)	0.16±0.05	0.04±0.01	0.31±0.10	0.09±0.02
Basal-apical (N)	0.11±0.04	0.08±0.02	0.20±0.09	0.19±0.05
Transverse/longitudinal force ratio				
LV	0.63±0.13	0.31±0.07	0.54±0.12	0.22±0.07
RV	1.55±0.42	0.69±0.19	1.64±0.54	0.48±0.18

Results are given as mean±SD (standard deviation) in all 23 subjects, using data with respiratory gating at 1.5T with manual LV and RV delineations.

### Reproducibility and sensitivity

**Fig 6:**
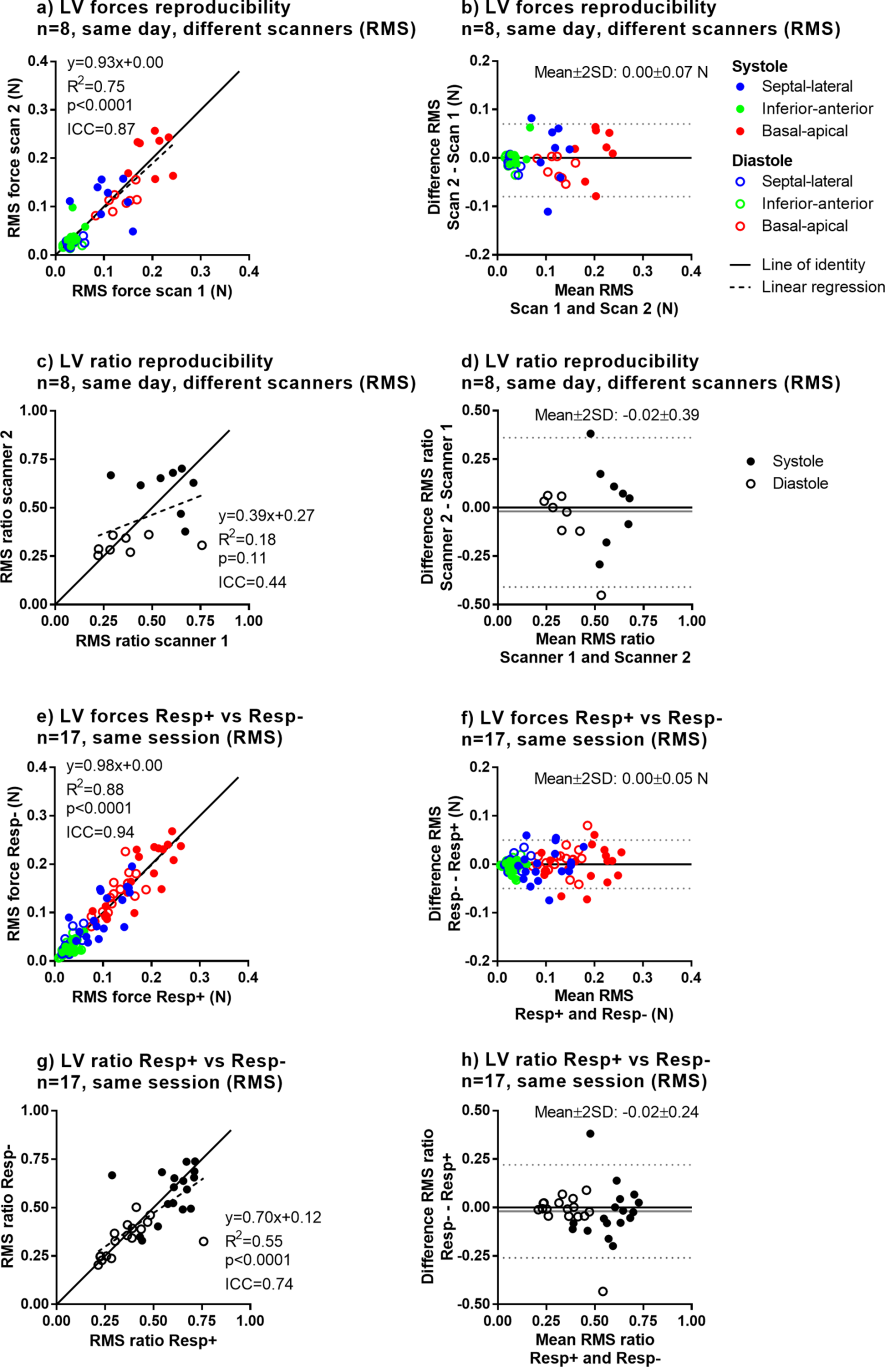
Reproducibility (different scanners, same day) and sensitivity to respiratory gating for LV forces and force ratios (RMS). Full results including comparisons of 1.5T vs 3T, scans on different days, influence of LV segmentation method as well as results for force peaks are shown in Table 4 and Supporting File S3 Appendix. Panels a) and b) show reproducibility of LV forces, and Panels b) and c) show reproducibility of force ratios. Panels e), f) g) and h) show sensitivity to respiratory gating.

**Fig 7:**
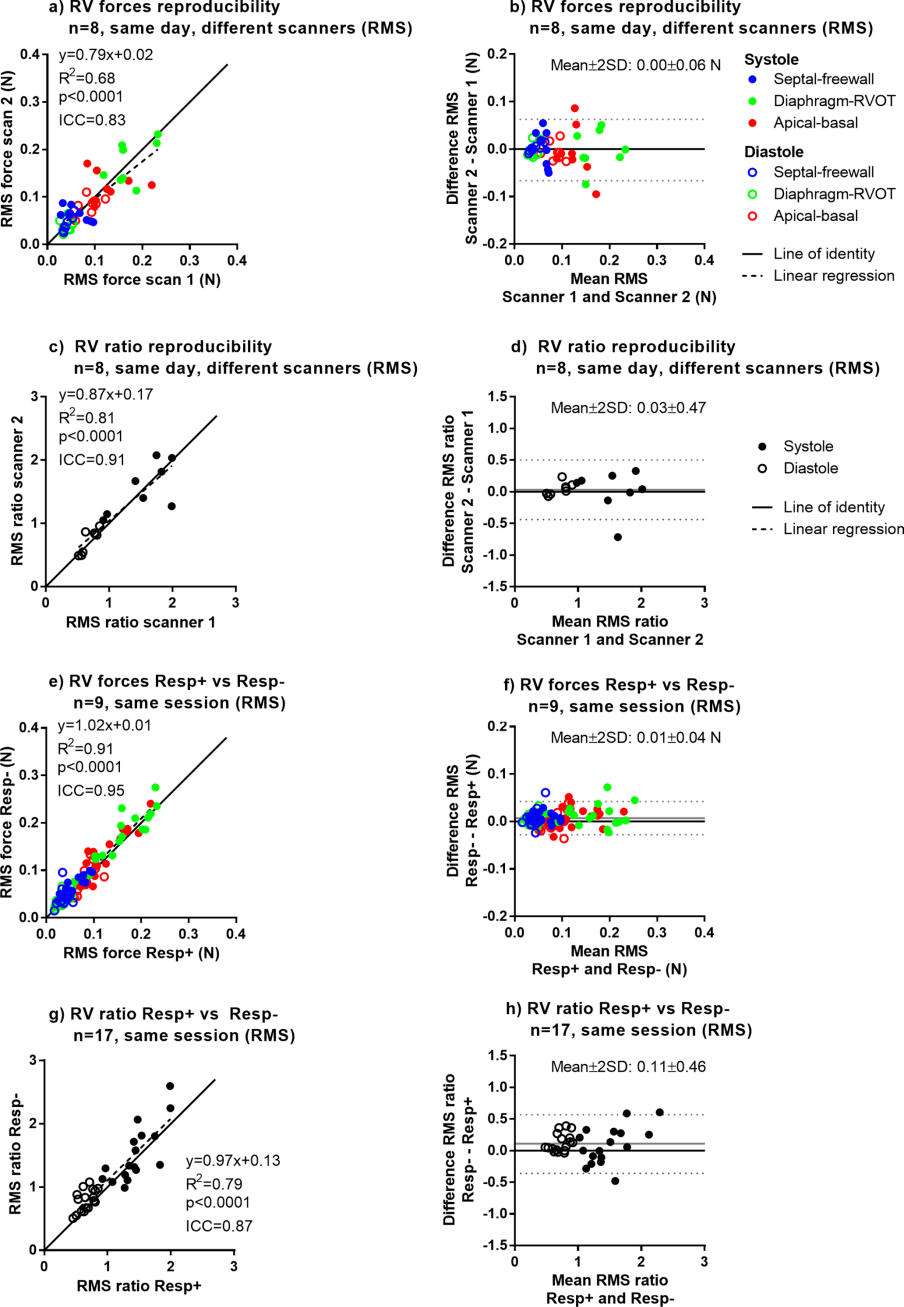
Reproducibility (different scanners, same day) and sensitivity to respiratory gating for RV forces and force ratios (RMS). Full results including comparisons of 1.5T vs 3T and scans on different days as well as results for force peaks are shown in Table 4 and Supporting File S4 Appendix. Panels a) and b) show reproducibility of RV forces, and Panels b) and c) show reproducibility of force ratios. Panels e), f) g) and h) show sensitivity to respiratory gating.

Table 4 summarizes all reproducibility and sensitivity results, for both the RMS and peak methods and for the LV and RV. The full dataset of force curves derived from 4D flow images and subject characteristics is available in Supporting File S2 Appendix. Graphical results are shown in Figs 6 and 7, and in S3 Appendix and S4 Appendix. Overall, ICC values were higher for RMS and peak forces compared to the force ratio (0.91±0.05 vs 0.80±0.16, p = 0.002). Short-term scan-rescan data on the same scanner showed strong to very strong agreement (ICC range 0.82–0.94), scans on different field strengths (1.5T vs 3T) showed strong to very strong agreement (ICC range 0.78–0.94), and comparing measurements based on LV segmentation performed manually and with the automatic method showed very strong agreement (ICC range 0.96–0.98).

**Table 4:** Reproducibility and sensitivity of hemodynamic forces and force ratio using RMS and peaks.

	Left ventricle				Right ventricle			
	LV RMS values (N)	LV Peak values (N)	LV RMS ratio	LV Peak ratio	RV RMS values (N)	RV Peak values (N)	RV RMS ratio	RV Peak ratio
Reproducibility (same day, different scanners, n = 8)								
Scan 1	0.09±0.07	0.19±0.13	0.47±0.18	0.37±0.15	0.09±0.06	0.18±0.10	1.12±0.54	0.98±0.55
Scan 2	0.09±0.07	0.18±0.14	0.45±0.17	0.36±0.20	0.09±0.05	0.18±0.10	1.14±0.52	0.99±0.71
Bias	0.00±0.04	-0.01±0.14	-0.02±0.19	-0.01±0.36	0.00±0.03	0.00±0.07	0.03±0.24	-0.02±0.65
ICC	0.87	0.88	0.44	0.49	0.83	0.78	0.91	0.85
Scan-rescan (different days, 1–12 days between, median 6 days, same scanner, n = 9)								
Scan 1	0.08±0.07	0.17±0.13	0.48±0.18	0.38±0.18	0.08±0.06	0.15±0.10	1.12±0.52	1.02±0.68
Scan 2	0.08±0.07	0.17±0.13	0.45±0.19	0.35±0.18	0.08±0.06	0.17±0.12	1.17±0.51	1.11±0.72
Bias	0.00±0.03	0.01±0.06	-0.03±0.11	-0.04±0.11	0.00±0.03	0.01±0.05	0.05±0.22	0.10±0.23
ICC	0.92	0.91	0.82	0.82	0.87	0.88	0.91	0.94
Field strengths (1.5T and 3T scanners, same day, random order, n = 6)								
1.5T	0.08±0.06	0.17±0.13	0.50±0.25	0.42±0.21	0.07±0.05	0.15±0.09	1.30±0.73	1.31±0.88
3T	0.08±0.06	0.17±0.13	0.50±0.23	0.45±0.26	0.07±0.05	0.14±0.09	1.29±0.78	1.25±0.94
Bias	0.00±0.02	0.00±0.05	0.00±017	0.03±0.15	0.00±0.02	-0.01±0.03	-0.01±0.36	-0.06±0.50
ICC	0.93	0.92	0.78	0.80	0.94	0.93	0.9	0.86
Respiratory gating (with and without respiratory gating, same scan session, n = 17)								
Resp+	0.08±0.07	0.18±0.13	0.48±0.17	0.37±0.16	0.08±0.06	0.16±0.10	1.05±0.46	0.94±0.54
Resp-	0.08±0.07	0.18±0.13	0.46±0.16	0.36±0.16	0.09±0.06	0.18±0.11	1.16±0.50	1.06±0.62
Bias	0.00±0.02	0.00±0.06	-0.02±0.12	-0.01±0.12	0.01±0.02	0.02±0.04	0.11±0.23	0.12±0.25
ICC	0.94	0.90	0.74	0.72	0.95	0.90	0.87	0.89
LV segmentation (using manual and automatic segmentation, n = 12)								
Manual	0.08±0.06	0.18±0.14	0.47±0.20	0.37±0.18	-	-	-	-
Automated	0.08±0.06	0.17±0.13	0.44±0.18	0.36±0.17	-	-	-	-
Bias	-0.01±0.01	-0.01±0.02	-0.03±0.04	-0.01±0.04	-	-	-	-
ICC	0.98	0.98	0.96	0.97	-	-	-	-

Graphical results are shown in Figs 6 and 7 and in S3 Appendix and S4 Appendix. Group values are given as mean±SD. ICC = intraclass correlation coefficient. -: not available.

Fig 6 shows reproducibility for LV hemodynamic forces, showing strong between-scanner reproducibility for forces (ICC = 0.87) and weak agreement for transverse/longitudinal ratio (ICC = 0.44). Scans with and without respiratory gating showed very strong agreement for forces (ICC = 0.94) and strong agreement for transverse/longitudinal ratio (ICC = 0.90).

Fig 7 shows reproducibility for RV hemodynamic forces, showing strong same-day reproducibility for forces (ICC = 0.83) and strong agreement for transverse/longitudinal ratio (ICC = 0.78). Scans with and without respiratory gating showed strong agreement for forces (ICC = 0.83) and very strong agreement for transverse/longitudinal ratio (ICC = 0.91).

**Fig 8:**
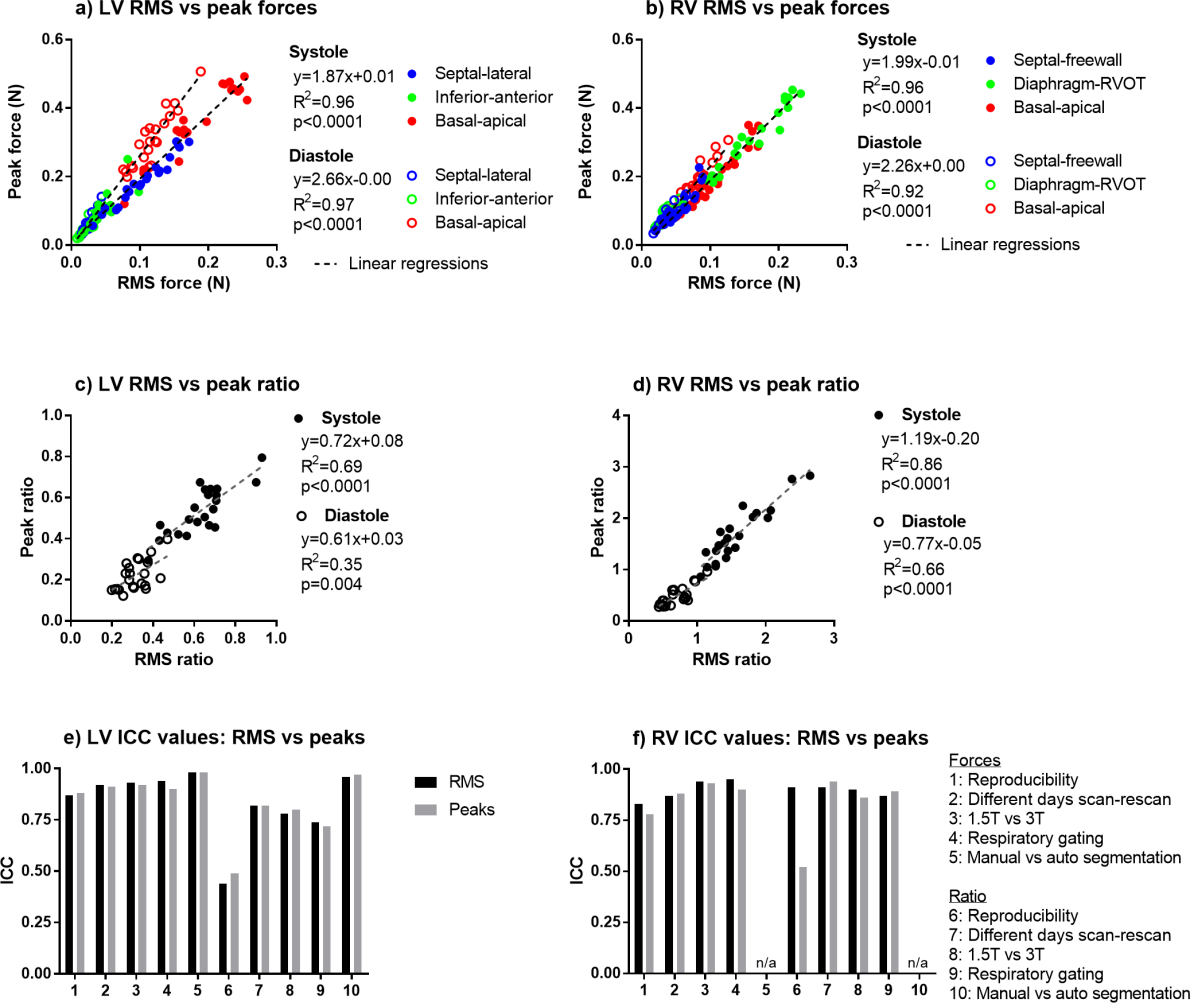
Comparison of RMS and peak forces and ratio of transverse/longitudinal forces. Panels a) and b) show comparison on of RMS and peak forces in the LV and RV, respectively. Linear regressions show strong to very strong correlations for systolic and diastolic forces. Panels c) and d) show comparison of the transverse/longitudinal ratio based on RMS and peak forces in the LV and RV respectively, with weak to strong correlations. Panels e) and f) show comparison of ICC agreement values for the LV and RV respectively. Legend to x-axis numbers is given in the right part of Panel f). There was no systematic difference in ICC between RMS and peak analysis, neither for the LV (0.84±0.16 vs. 0.84±0.15, p = 0.99), nor for the RV (0.80±0.30 vs. 0.74±0.31, p = 0.21).

Fig 8 shows a comparison of RMS and peak forces in LV and RV. Associations between RMS and peak force measurements were very strong for the LV (systole: R^2^ = 0.96, diastole: R^2^ = 0.97) and RV (systole: R^2^ = 0.96, diastole: R^2^ = 0.92). The LV force ratio was larger for the RMS method compared to peaks in systole (0.63±0.14 vs 0.54±0.12, p<0.0001) and diastole (0.31±0.07 vs 0.22±0.07, p<0.0001), with a moderate association in systole (R^2^ = 0.69) and weak association in diastole (R^2^ = 0.35). For the RV ratio no difference was found in systole (1.55±0.41 vs 1.64±0.54, p = 0.07), while RMS and peak ratios differed in diastole (0.69±0.19 vs 0.48±0.18, p<0.0001), with a strong association in systole (R^2^ = 0.86) and moderate in diastole (R^2^ = 0.66). There was no systematic difference in ICC between RMS and peak analysis for forces, neither for the LV (0.84±0.16 vs. 0.84±0.15, p = 0.99), nor for the RV (0.80±0.30 vs. 0.74±0.31, p = 0.21). Similarly, there was no difference in ICC between RMS and peak analysis for the force ratio (0.81±0.16 vs 0.78±0.17, p = 0.95).

## Discussion

The results of this study show high reproducibility for LV and RV hemodynamic forces, while the transverse/longitudinal force ratio is less reproducible. Scans with and without respiratory gating showed high agreement. Furthermore, good agreement was found between field strengths, and between different LV segmentation methods. Phantom validation shows good agreement between 4D flow MRI and PIV laser measurements. Software for hemodynamic force analysis is provided free, for research use, including source code.

### Accuracy and precision

The good agreement in the phantom validation shows that hemodynamic forces can be quantified using 4D flow MRI, even though the quantification method involves differentiation of the data which in general is sensitive to noise and low resolution. However, the spatial and temporal resolution of 4D flow MRI appears to be sufficient for calculation of hemodynamic forces, although a small underestimation remains. The force ratio was not investigated in the phantom setup. This is motivated by the axisymmetric flow conditions in vortex ring formation in a circular nozzle, leading to a transverse force that is exactly zero in all timeframes. To further study the force ratio in vitro, a new asymmetric phantom setup must be developed.

The generally strong to very strong agreements between different measurement conditions found in this study reinforce the validity and robustness of the method. However, while agreement is generally good on a group basis, some variability remains, limiting the use of hemodynamic forces computed from current 4D flow methods in single patients. Therefore, forces should be viewed as reproducible primarily on a group average basis. The transverse/longitudinal force ratio showed weaker reproducibility in general, which may be caused by a compound effect of uncertainty in the forces in the nominator and denominator in the ratio computation. This suggests that comparisons of hemodynamic forces, and especially the transverse/longitudinal ratio are best performed on a group basis, although further developments of the hemodynamic forces analysis method and measurement protocol may provide more precise ratio measurements. Furthermore, the high reproducibility for forces and lower reproducibility for transverse/longitudinal force ratios suggests that the forces may be robust with respect to small changes in the measurement and analysis protocol, while additional care must be taken for force ratio quantification, especially in the LV where weak agreement was found between scanners (ICC = 0.44). This shows that LV force ratio data can not be pooled between scanners when doing larger studies, and all subjects should therefore be scanned on the same scanner. This is reinforced by the strong repeatability for the RV ratio when using the same scanner on different days (ICC = 0.82). Notably, our recent study on hemodynamic force physiology used data from a single scanner only [3].

The strong correlation between RMS and peak force measurements was not expected a priori, but can be explained by similar curve shapes between the subjects. When comparing RMS and peak analysis, we found similar agreement for both methods in the LV and RV, suggesting that peak or RMS values can be used as required by the physiological or clinical application.

The smaller transverse/longitudinal ratio computed from peaks compared to RMS can be explained by differences in the definition of the two measures. The RMS ratio used by Arvidsson et al. [3] includes both transverse force components in the numerator (septal-lateral and inferior-anterior in the LV, diaphragm-RVOT and septal-freewall in the RV). In contrast, the peak ratio used by Eriksson et al. [2,4] only includes the main transverse force component in the numerator (septal-lateral in the LV, diaphragm-RVOT in the RV), which intrinsically leads to a smaller ratio.

### Relation to earlier studies

The results in this study are in line with previous data for diastole in healthy volunteers reported by Eriksson et al. [2] (basal-apical forces: 0.31±0.08 vs 0.25±0.10 N, septal-lateral forces: 0.07±0.03 vs 0.08±0.04, ratio: 0.22±0.07 vs 0.23±0.12). The present findings extend previous results by [2–4] demonstrating a high reproducibility of hemodynamic forces and good agreement between scans with and without respiratory gating, scans on different days, and using different field strengths. Previous studies have also investigated the spatial distribution of pressure at different sites inside the LV. In a canine model, Courtois et al. [5] found a significant early diastolic pressure gradient along the LV inflow tract with minimum pressure in the apex, suggesting suction of the blood toward the LV apex. This pressure gradient was reversed at peak E-wave, so that apical pressure exceeded atrial pressure. Interestingly, the acceleration (E-wave upslope) pressure gradient was larger compared to the deceleration gradient (E-wave downslope), contrary to our findings where the late E-wave force was stronger. This may be explained by the limited spatial resolution of 4D flow MR, since the apical LV lumen is small at the onset of the E-wave (end of systole).

A related method, first applied by Ebbers et al. in healthy volunteers [27,28], uses 4D flow data to compute pressure differences inside the heart [17,27–31]. While the pressure difference method is similar to hemodynamic forces, some important differences exist. Eriksson et al. [31] quantify pressure differences by first computing pressure gradients from 4D flow, then computing the pressure difference field using the pressure Poisson equation (PPE) and finally studying local differences in the resulting pressure field. In contrast, the hemodynamic force method computes the force in the whole LV directly from 4D flow data without the PPE computation. This is beneficial, since solving the PPE is time-consuming and may give inaccurate results unless advanced algorithms are used [17,18]. Although the PPE method is more complex than the one presented here, it has an advantage in giving local pressure differences within the LV. Conversely, the advantage of hemodynamic forces is that it gives a single force for the whole LV, separated into three spatial components to enable a more intuitive physiological interpretation. Therefore, both methods complement each other and the choice of method may depend on the specific application.

Two previous studies have used different conventions for the force ratio, with the main difference being whether to put the basal-apical force in the numerator or in the denominator (and the transverse forces in the denominator or numerator, accordingly) [2–4]. If the transverse forces (which are usually small) are used in the denominator, a small error in the transverse force may lead to a large change in the ratio. Conversely, using the larger basal-apical values in the denominator may lead to a less error-sensitive ratio measure and we therefore opted for this choice.

### Physiological and pathophysiological aspects

The basal-apical force pattern found in this study during the early rapid filling phase of the LV is in line with a previous study by Courtois et al. [6], who used dual-head pressure transducers to measure LV diastolic pressure gradients in dogs. They found a lower pressure in the apex during the first half of early rapid filling compared to a reading 3 cm more basally, and a reversed gradient during the second half. This is in agreement with the brief negative apical-basal hemodynamic force found right after end-systole and subsequent reversal into a positive apical-basal force during early rapid filling.

Hemodynamic force measurements may be used to extend our knowledge about normal physiology of the four-chambered heart including atrioventricular and LV-RV interactions [32,33], and may further elucidate the functional differences between the LV as a pressure pump and the RV as a volume pump [34,35]. Analysis of LV hemodynamic force dynamics may also be combined with transverse and longitudinal impedance quantification [36] to further advance our understanding about normal physiology and pathophysiology. Further avenues for exploration include altered cardiovascular states such as pregnancy, interaction between the RV and pulmonary vasculature and disease states including congenital heart disease and pericardial diseases.

During contraction and relaxation of the LV, there is a rotational motion of the myocardium called torsion [37] resulting from the spatial arrangement of cardiomyocytes [38,39]. Since blood flow follows from myocardial dynamics, any torsional motion of the myocardium will be reflected in the blood flow, and subsequently included in the hemodynamic force measurement through the 4D flow acquisition. Therefore, torsion effects are included in the data presented here, but at the time of writing we had no method for discriminating how different aspects of the ventricular contraction (longitudinal, circumferential, radial, torsion) influences hemodynamic forces. The physiological and pathophysiological interplay between hemodynamic forces and LV torsion remains to be investigated.Limitations.

The present study should be viewed in light of its limitations. The hemodynamic forces method is based on the Navier-Stokes equations for Newtonian, laminar flow without turbulence modelling. Furthermore, small-scale fluctuations leading to energy dissipation [40] is not captured by 4D flow MRI measurements. This means that turbulent flows, e.g. in severe mitral stenosis or aortic regurgitation with jet formation may need special turbulence measurement methods [41] to give accurate results. Additionally, taking into account non-Newtonian blood flow effects [42] may be an interesting avenue of future research.

Our healthy subjects had high end-diastolic volumes (175±36 ml, range 112–247 ml). However, normalized for body surface area (BSA), the cardiac dimensions of subjects in this study fall only slightly outside previously published normal ranges [43,44].Finally, the study population is young and healthy and may not fully represent reproducibility conditions in older patients.

## Conclusions

Hemodynamic forces measured in the LV and RV using 4D flow MRI show good accuracy with a small underestimation compared to laser-based particle image velocimetry, high reproducibility and good agreement between scans with and without respiratory gating, for different field strengths, and for different LV segmentation methods. Transverse/longitudinal force ratio quantification shows weaker agreement, suggesting that comparison of ratio values should be performed with care. Software for hemodynamic force analysis is provided free, for research use, including source code.

## Supporting information

S1 Appendix Software instructions. Instructions for download and use of freely available software for hemodynamic force analysis, including open source code.
(PDF)

S2 Appendix Complete data set. Microsoft Excel files (.xlsx) containing the full data set of hemodynamic forces derived from 4D flow data in the LV and RV in all subjects (n = 23).
(ZIP)

S3 Appendix LV graphs. Graphical results for reproducibility of left ventricular (LV) hemodynamic force measurements.
(PDF)

S4 Appendix RV Graphs. Graphical results for reproducibility of right ventricular (RV) hemodynamic force measurements.
(PDF)

S1 Movie Hemodynamic forces visualization. Animation showing the hemodynamic force vector (white arrow) and the relative pressure field (color scale) in a healthy subject (cf. Fig 4).
(MPG)
