# Supplementary material for: Hemodynamic forces in the left and right ventricles of the human heart using 4D flow magnetic resonance imaging: Phantom validation, reproducibility, sensitivity to respiratory gating and free analysis software
Source: PLoS One. 2018 Apr 5;13(4):e0195597. doi: 10.1371/journal.pone.0195597 (PMC5886587; doi:10.1371/journal.pone.0195597)
Supplement: S1 Appendix — Instructions for download and use of freely available software for hemodynamic force analysis, including open source code. (PDF) [file pone.0195597.s001.pdf]

S1 Appendix: Software for hemodynamic force quantification in 4D flow data

Hemodynamic forces in the left and right ventricles of the human heart using 4D flow magnetic resonance imaging: reproducibility and sensitivity to respiratory gating, field strength and ventricle segmentation, with free analysis software

Johannes Töger[1], Per M Arvidsson[1], Jelena Bock[1], Mikael Kanski[1],
Gianni Pedrizzetti[2], Marcus Carlsson[1], Håkan Arheden[1], Einar Heiberg[1,3*]

[1]Lund University, Skane University Hospital, Department of Clinical Physiology, Lund, Sweden
[2]Department of Engineering and Architecture, University of Trieste, Trieste, Italy
[3]Department of Biomedical Engineering, Faculty of Engineering, Lund University, Lund, Sweden

PLOS One 2018, doi: 10.1371/journal.pone.0195597

*: Corresponding author: Einar Heiberg
Department of Clinical Physiology, Lund University Hospital, SE-22185 Lund, Sweden
einar.heiberg@med.lu.se
Phone: +46-46-171605, Fax: +46-46-151769

Technical questions on hemodynamic forces toolkit code should be directed to Johannes Töger (johannes.toger@med.lu.se).

Contents

Introduction and terms of use 2

Installation 3

Instructions for use 3

Plug-in menu items 8

Code overview 9

References 10

Introduction and terms of use

This Supporting File describes how to install and use the software for hemodynamic force quantification provided with the parent article (1). The software is provided for free use for research purposes, provided that the relevant articles for Segment (2) and hemodynamic forces (1, 3) are cited in conference talks and publications.

If the methods for relative pressure field computation are used, the relevant publications describing the method should be cited (4, 5). The relative pressure computation code (`antigradient2.c` and `antigradient2.m`) is distributed with Segment under the MIT license, courtesy of Gunnar Farnebäck. The `antigradient2` code is part of the spatial domain toolbox, available at https://github.com/GunnarFarneback/spatial_domain_toolbox.

Technical questions on the hemo-dynamic forces code should be directed to Johannes Töger (johannes.toger@med.lu.se).

Installation

MATLAB R2014a or later is required to run the software. First, download the Segment source code from https://github.com/Cardiac-MR-Group-Lund/segment-open. Thereafter, you can obtain the hemodynamic forces plug-in source code by contacting Johannes Töger (johannes.toger@med.lu.se) or Einar Heiberg (einar.heiberg@med.lu.se). Extract the source code into an empty directory, navigate to that directory in MATLAB and type 'segment' and press enter to start. A 4D flow dataset including all required images to quantify hemodynamic forces is available for download through the FourFlow project (http://fourflow.heiberg.se) at the link http://fourflow.heiberg.se/downloads/example_data_hemodynamic_forces.mat.

Instructions for use

First, download, install and start Segment as described above.

1. Load image data, either from your own DICOM MRI data or from the provided file example_data_hemodynamic_forces.mat (see above). You should then have the following
2. datasets loaded. <u>If you load the example .mat file, you can skip steps 2, 3 and 4.</u>
    a. 4D flow (magnitude stack + 3 flow stacks)
    b. Cine long-axis images (2ch, 3ch, 4ch)
    c. Cine short-axis images with LV and RV segmentations
3. Perform delineation of left or right ventricle (LV or RV) in all slices and time frames
    a. Segment includes a semi-automatic tool for LV delineation (6). Instructions for this process can be found in the Segment manual, available in the Docs folder of the Segment installation.
    b. For RV, manual delineation is currently the only available option.
4. *Important:* Add annotations for atrioventricular plane (AV-plane) in end-diastole using the annotation point tool under the 'Misc' part of the toolbox in the lower right corner (Figure 1). The points must be named "AV plane" to be recognized later in the process.

5. *Important:* For each long-axis and short-axis image stack, set the correct image view plane by right-clicking each thumbnail in the leftmost part of the Segment window and clicking "Set image description". In the window that appears, set the correct view plane in the middle listbox. For example, the 3-chamber image should be set to "3CH".

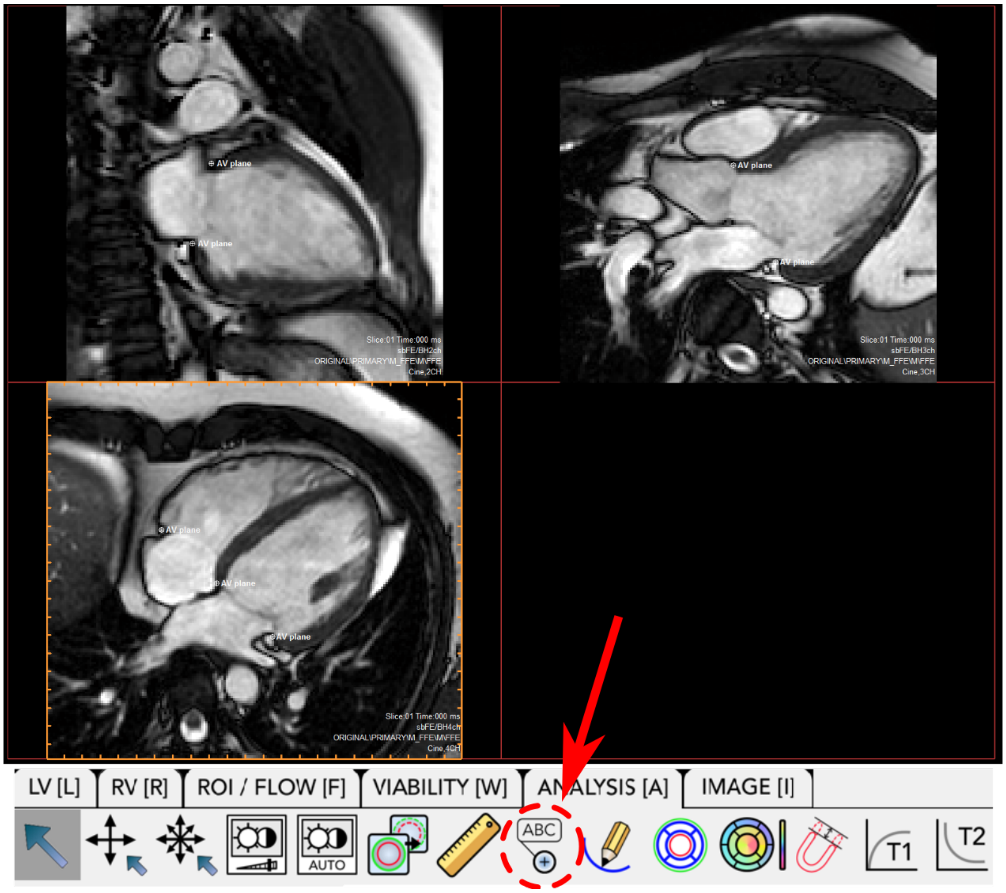

Figure 1: Definition of AV plane points using the annotation point tool.

6. Launch the hemodynamic forces GUI in the menu bar: Plug-ins → Hemodynamic Forces → Quantification GUI. The GUI is shown in Figure 2. The most important elements are the following:
    a. View of the short-axis stack. The LV or RV delineation should be visible here, depending on what is selected in the segmentation selector (f).
    b. View of the long-axis stack. The 4-chamber image should be shown here.
    c. Results window. Hemodynamic force curves will appear here.
    d. Sliders for timeframe (long-axis and short-axis) and slice (short-axis).
    e. Hemodynamic force coordinate system/direction adjustment. The 'auto' buttons should be used for reproducibility. Use the 'flip' buttons to rotate the direction 180 degrees (sometimes needed after 'auto'). Manual changes can be performed by clicking and dragging in panels a) and b). The center points can also be dragged. Moving the center points is only for visualization purposes, and does not influence the quantification process.

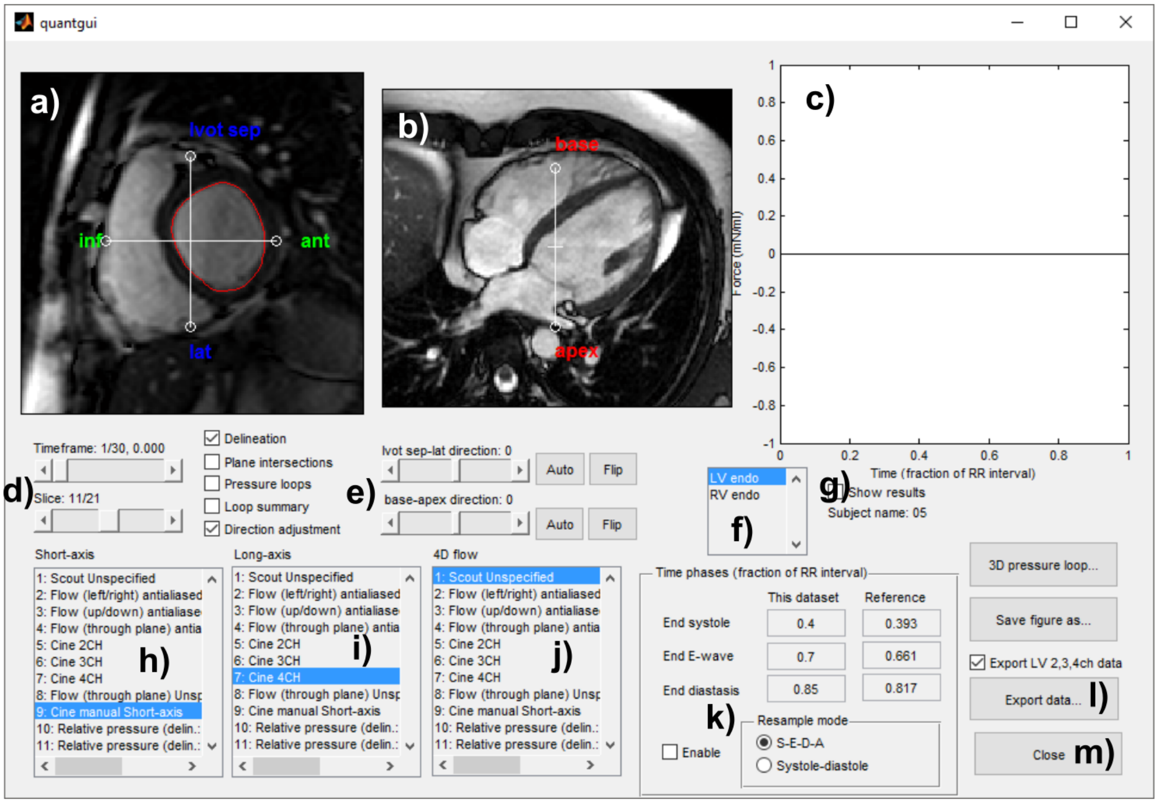

Figure 2: Quantification GUI immediately after launch with the example file loaded, showing annotations of important elements. See text for details.

f. LV or RV segmentation selector.

g. Show results checkbox. When this is checked, the resulting hemodynamic force graphs are shown in panel c).

h. Short-axis stack selector. If the short-axis stack is correctly identified in step 4, this should be automatically detected. Stack name in example data file: "9: Cine manual short-axis".

i. Long-axis stack selector. If the 4CH stack is correctly identified in step 4, this should be automatically detected. If not, choose the 4CH stack here. Stack name in example data file: "7: Cine 4CH".

j. 4D flow stack selector. This should be automatically detected if the 4D flow is correctly loaded. Stack name in example data file: "1: Scout unspecified".

k. Time phase resampling to a reference heartbeat. All values are given as a fraction of the R-R interval (7).

l. Export data – will copy data to the clipboard. This can then be pasted into Excel.

m. Close button – to exit the GUI.

7. To perform hemodynamic force quantification, perform the following steps in the GUI. The expected result is given in Figure 3.

   a. Check that all stacks (short-axis, long-axis 4ch, 4D flow) are correctly set in panels h, i, and j in figure 2.

   b. Set reference directions using the 'auto' and 'flip' buttons (e).

   c. Choose the desired segmentation in (f).

   d. Check the 'Show results' checkbox (g).

   e. Optional: Enter cardiac phase timing information in the "Time phases" box (k) and check the box to enable resampling to a common time axis. The exported data will contain both resampled and non-resampled data.

   f. To export data to the clipboard, click the 'Export data' button (l).

   g. Paste the results (Ctrl-V) into Excel.

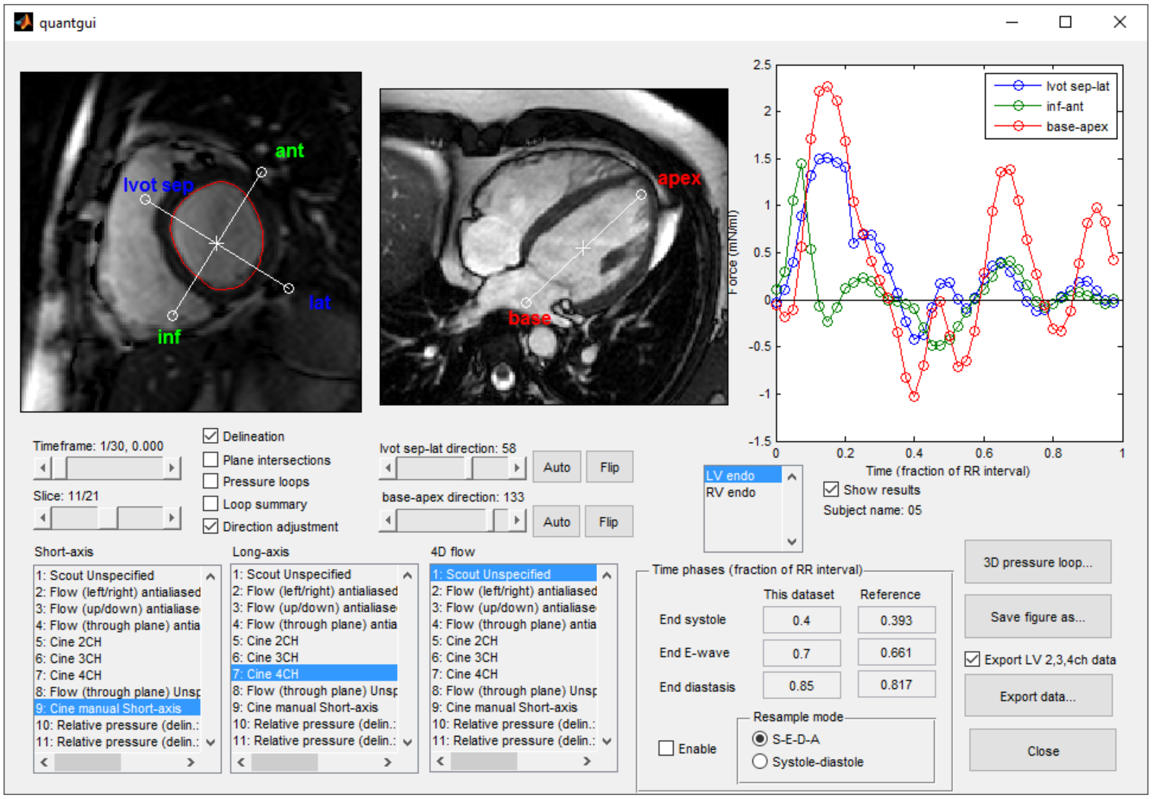

Figure 3: Quantification GUI with all settings correct for hemodynamic force quantification in this selected subject.

Plug-in menu items

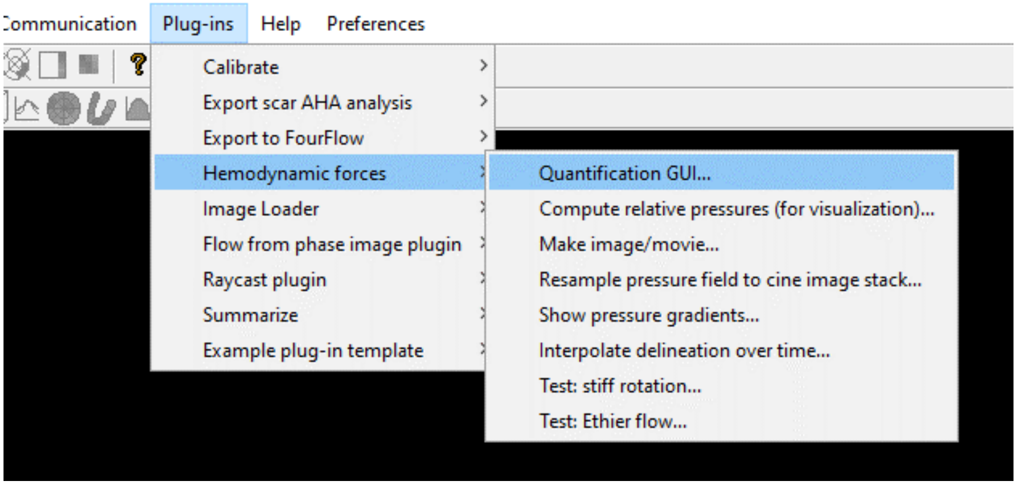

Quantification GUI...	Main hemodynamic forces quantification GUI. For instructions see above.
Compute relative pressures (for visualization)...	Computes relative pressure field. Requires 4D flow stack and segmentation.
Make image/movie...	Pressure field overlay on top of cine images
Resample pressure field to cine image stack...	Resamples pressure field data (computed using above function) from 4D flow geometry to any stack geometry.
Show pressure gradients...	Display pressure gradients for debugging purposes.
Interpolate delineation over time...	Given LV or RV segmentation in a few time phases, interpolate over time. Manual corrections will be needed, especially in the basal part of LV and RV. For accurate LV segmentations, please use the built-in tool in Segment (6).
Test: stiff rotation...	Test the relative pressure field calculation with a numerical rotating phantom
Test: Ethier flow...	Test the relative pressure field calculation with an exact 3D+T Navier-Stokes solution (8)

Code overview

All code is written in MATLAB. The exception is the code for multigrid computation of relative pressure fields, which is written in C by Ebbers and Farneback (4, 5). The code is contained in the file `plugin_hemoforce.m` and the directory `+hemoforce`, with three auxiliary functions in the directory `+lcs2d`. The main hemodynamic force quantification code is located in the file `+hemoforce/quantgui.m`.

List of files as of Segment v2.0R5436

`plugin_hemoforce.m`	Plugin file, registers menu items in Segment on startup.
`+hemoforce/antigradient2.c`	C code for multigrid solver (4, 5) used in relative pressure field calculation.
`+hemoforce/antigradient2.m`	Placeholder M-file for multigrid solver (4, 5), contains basic documentation
`+hemoforce/antigradient2.mexa64`	Linux 64-bit MEX-file for multigrid solver (4, 5)
`+hemoforce/antigradient2.mexw64`	Windows 64-bit MEX-file for multigrid solver (4, 5)
`+hemoforce/computegradients.m`	Auxiliary function for computing pressure gradient from 4D flow using Navier-Stokes equations
`+hemoforce/computegradients2d.m`	As above, but for the 2D case
`+hemoforce/computegradientsfrompressurefield.m`	Auxiliary function to compute pressure gradient from a computed pressure field
`+hemoforce/computepressure.m`	Compute pressure from 4D flow data loaded into Segment
`+hemoforce/interpolatedelineation.m`	Interpolation of LV/RV delineations
`+hemoforce/makeim.fig`<br>`+hemoforce/makeim.m`	Create cine/pressure overlay image
`+hemoforce/makemask_helper.m`	Auxiliary function for creating a binary mask from LV/RV delinations
`+hemoforce/quantgui.fig`<br>`+hemoforce/quantgui.m`	Main hemodynamic force quantification GUI
`+hemoforce/resampletostack.m`	Resample pressure field to cine geometry
`+hemoforce/runcompute.m`	Wrapper script to run pressure field calculations
`+hemoforce/showgradp.fig`<br>`+hemoforce/showgradp.m`	GUI for displaying pressure gradients for debug purposes
`+hemoforce/testethier.m`<br>`+hemoforce/testethier.mw`<br>`+hemoforce/testrotation.m`	Numerical test cases for pressure calculations
`+lcs2d/clamp.m`	Auxiliary function for colorspace transformations
`+hemoforce/xyz2xyz.m`<br>`+lcs2d/getgeometryfromsegment.m`<br>`+lcs2d/resamplestack.m`	Auxiliary functions for stack geometry transformations

References

1. Töger J, Arvidsson P, Bock J, et al.: Hemodynamic forces in the left and right ventricles of the human heart using 4D flow magnetic resonance imaging: reproducibility and sensitivity to respiratory gating, field strength and ventricle segmentation. 2017.

2. Heiberg E, Sjögren J, Ugander M, Carlsson M, Engblom H, Arheden H: Design and validation of Segment - freely available software for cardiovascular image analysis. *BMC Med Imaging* 2010; 10:1.

3. Arvidsson PM, Töger J, Carlsson M, et al.: Left and right ventricular hemodynamic forces in healthy volunteers and elite athletes assessed with 4D flow magnetic resonance imaging. *Am J Physiol - Hear Circ Physiol* 2016:ajpheart.00583.2016.

4. Farnebäck G, Rydell J, Ebbers T, Andersson M, Knutsson H: Efficient computation of the inverse gradient on irregular domains. *Proc IEEE Int Conf Comput Vis* 2007.

5. Ebbers T, Farnebäck G: Improving computation of cardiovascular relative pressure fields from velocity MRI. *J Magn Reson Imaging* 2009; 30:54–61.

6. Tufvesson J, Hedström E, Steding-Ehrenborg K, Carlsson M, Arheden H, Heiberg E: Validation and Development of a New Automatic Algorithm for Time-Resolved Segmentation of the Left Ventricle in Magnetic Resonance Imaging. *Biomed Res Int* 2015:970357.

7. Carlsson M, Heiberg E, Toger J, et al.: Quantification of left and right ventricular kinetic energy using four-dimensional intracardiac magnetic resonance imaging flow measurements. *Am J Physiol Heart Circ Physiol* 2012; 302:H893-900.

8. Ethier C, Steinman D: Exact fully 3D Navier-Stokes solutions for benchmarking. *Int J Numer Methods Fluids* 1994; 19:369–375.
