# Supplementary material for: Hemodynamic forces in the left and right ventricles of the human heart using 4D flow magnetic resonance imaging: Phantom validation, reproducibility, sensitivity to respiratory gating and free analysis software
Source: PLoS One. 2018 Apr 5;13(4):e0195597. doi: 10.1371/journal.pone.0195597 (PMC5886587; doi:10.1371/journal.pone.0195597)
Supplement: S3 Appendix — Graphical results for reproducibility of left ventricular (LV) hemodynamic force measurements. (PDF) [file pone.0195597.s003.pdf]

S3 Appendix: Graphical results for reproducibility of LV hemodynamic force measurements

Hemodynamic forces in the left and right ventricles of the human heart using 4D flow magnetic resonance imaging: reproducibility and sensitivity to respiratory gating, field strength and ventricle segmentation, with free analysis software

Johannes Töger[1], Per M Arvidsson[1], Jelena Bock[1], Mikael Kanski[1],

Gianni Pedrizzetti[2], Marcus Carlsson[1], Håkan Arheden[1], Einar Heiberg[1,3*]

[1]Lund University, Skane University Hospital, Department of Clinical Physiology, Lund, Sweden

[2]Department of Engineering and Architecture, University of Trieste, Trieste, Italy

[3]Department of Biomedical Engineering, Faculty of Engineering, Lund University, Lund, Sweden

PLOS One 2018, doi: 10.1371/journal.pone.0195597

*: Corresponding author: Einar Heiberg

Department of Clinical Physiology, Lund University Hospital, SE-22185 Lund, Sweden

einar.heiberg@med.lu.se

Phone: +46-46-171605, Fax: +46-46-151769

LV RMS forces

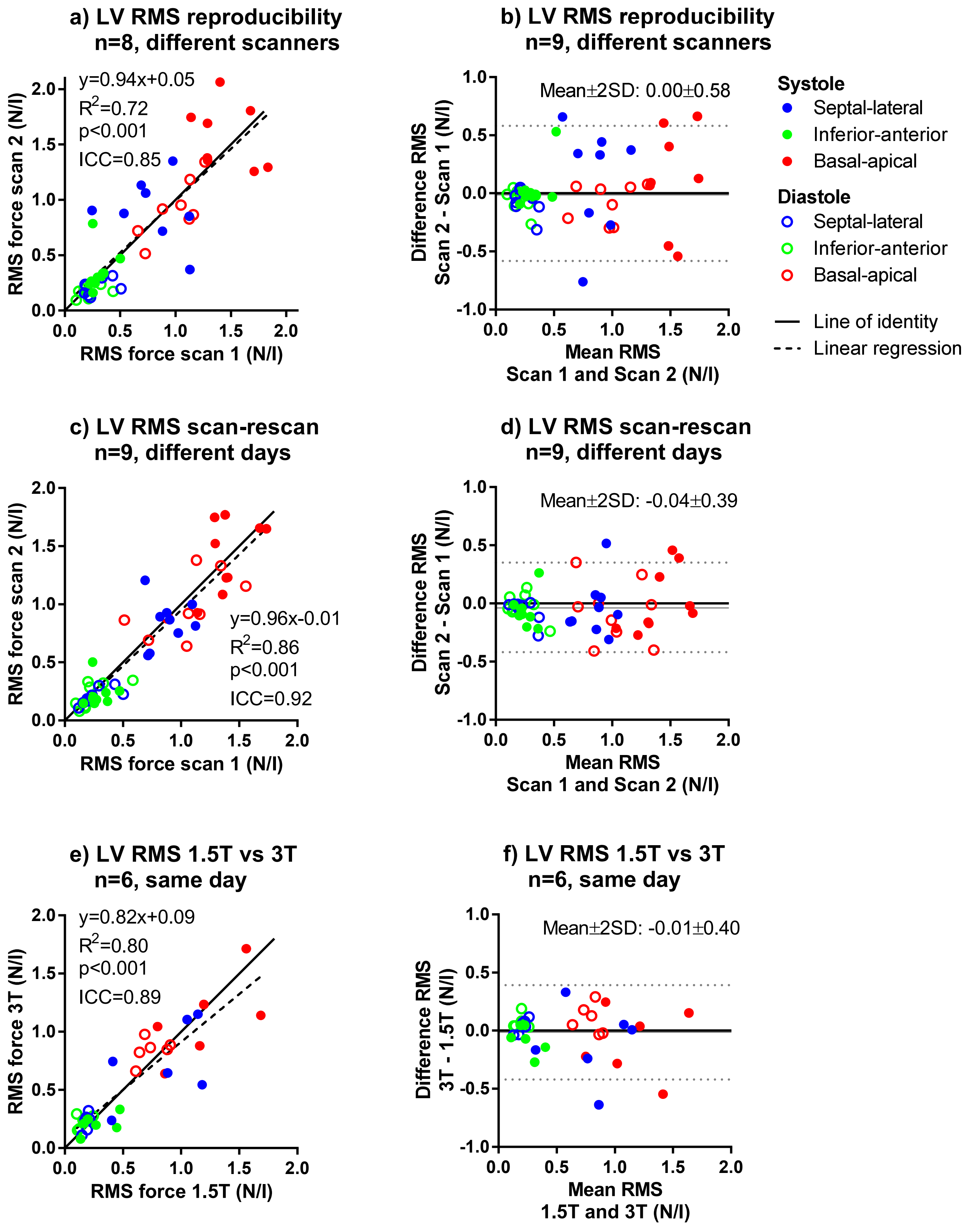

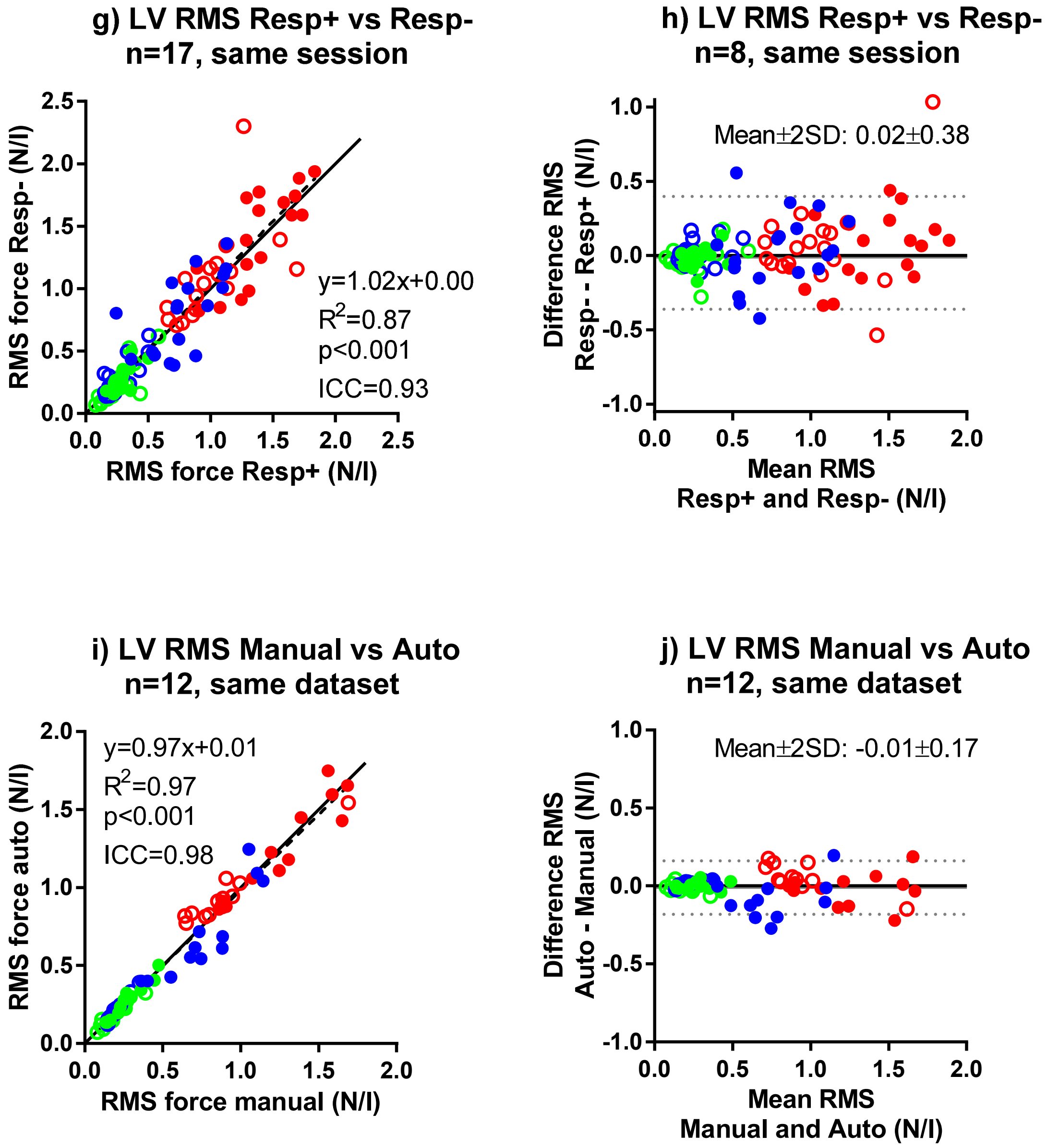
g) LV RMS Resp+ vs Resp-
n=17, same session
RMS force Resp- (N/l)
RMS force Resp+ (N/l)
y=1.02x+0.00
R2=0.87
p<0.001
ICC=0.93
h) LV RMS Resp+ vs Resp-
n=8, same session
Mean±2SD: 0.02±0.38
Difference RMS
Resp- - Resp+ (N/l)
Mean RMS
Resp+ and Resp- (N/l)
i) LV RMS Manual vs Auto
n=12, same dataset
y=0.97x+0.01
R2=0.97
p<0.001
ICC=0.98
RMS force auto (N/l)
RMS force manual (N/l)
j) LV RMS Manual vs Auto
n=12, same dataset
Mean±2SD: -0.01±0.17
Difference RMS
Auto - Manual (N/l)
Mean RMS
Manual and Auto (N/l)

LV peak forces

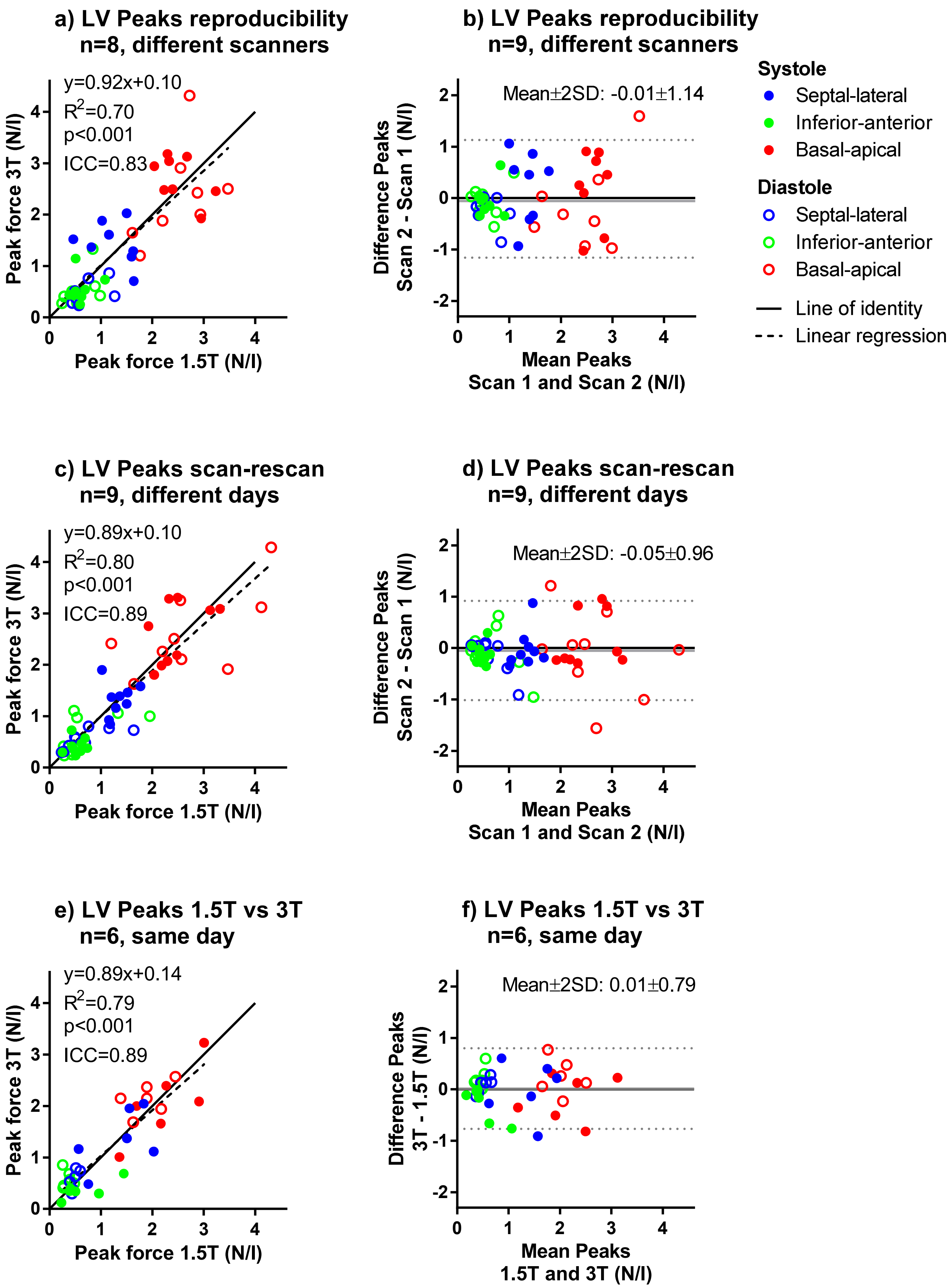

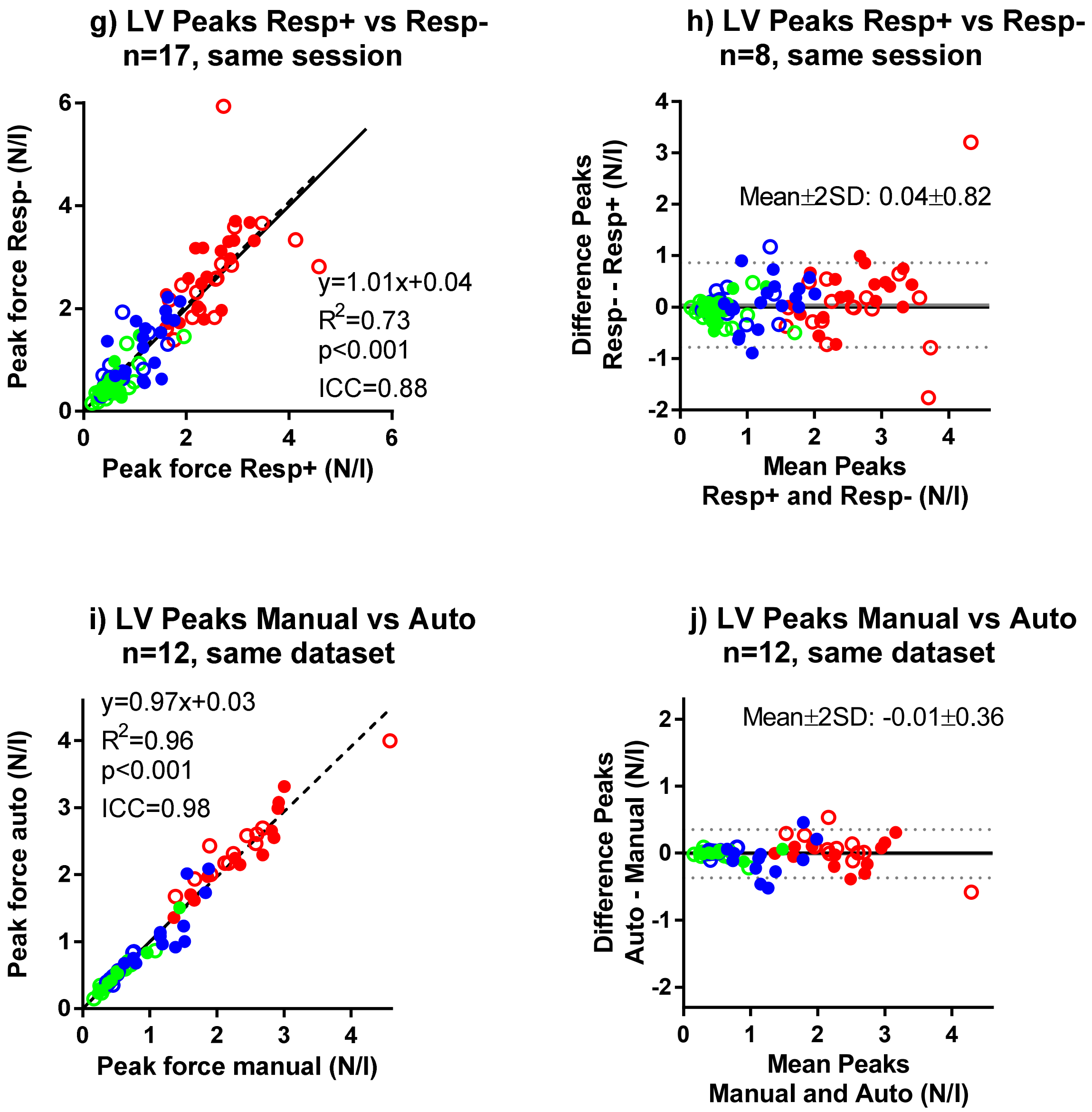
g) LV Peaks Resp+ vs Resp-
n=17, same session
Peak force Resp- (N/l)
Peak force Resp+ (N/l)
y=1.01x+0.04
R2=0.73
p<0.001
ICC=0.88
0
2
4
6
h) LV Peaks Resp+ vs Resp-
n=8, same session
Difference Peaks
Resp- - Resp+ (N/l)
Mean±2SD: 0.04±0.82
Mean Peaks
Resp+ and Resp- (N/l)
-2
-1
0
1
2
3
4
i) LV Peaks Manual vs Auto
n=12, same dataset
Peak force auto (N/l)
Peak force manual (N/l)
y=0.97x+0.03
R2=0.96
p<0.001
ICC=0.98
j) LV Peaks Manual vs Auto
n=12, same dataset
Difference Peaks
Auto - Manual (N/l)
Mean±2SD: -0.01±0.36
Mean Peaks
Manual and Auto (N/l)

LV RMS ratio

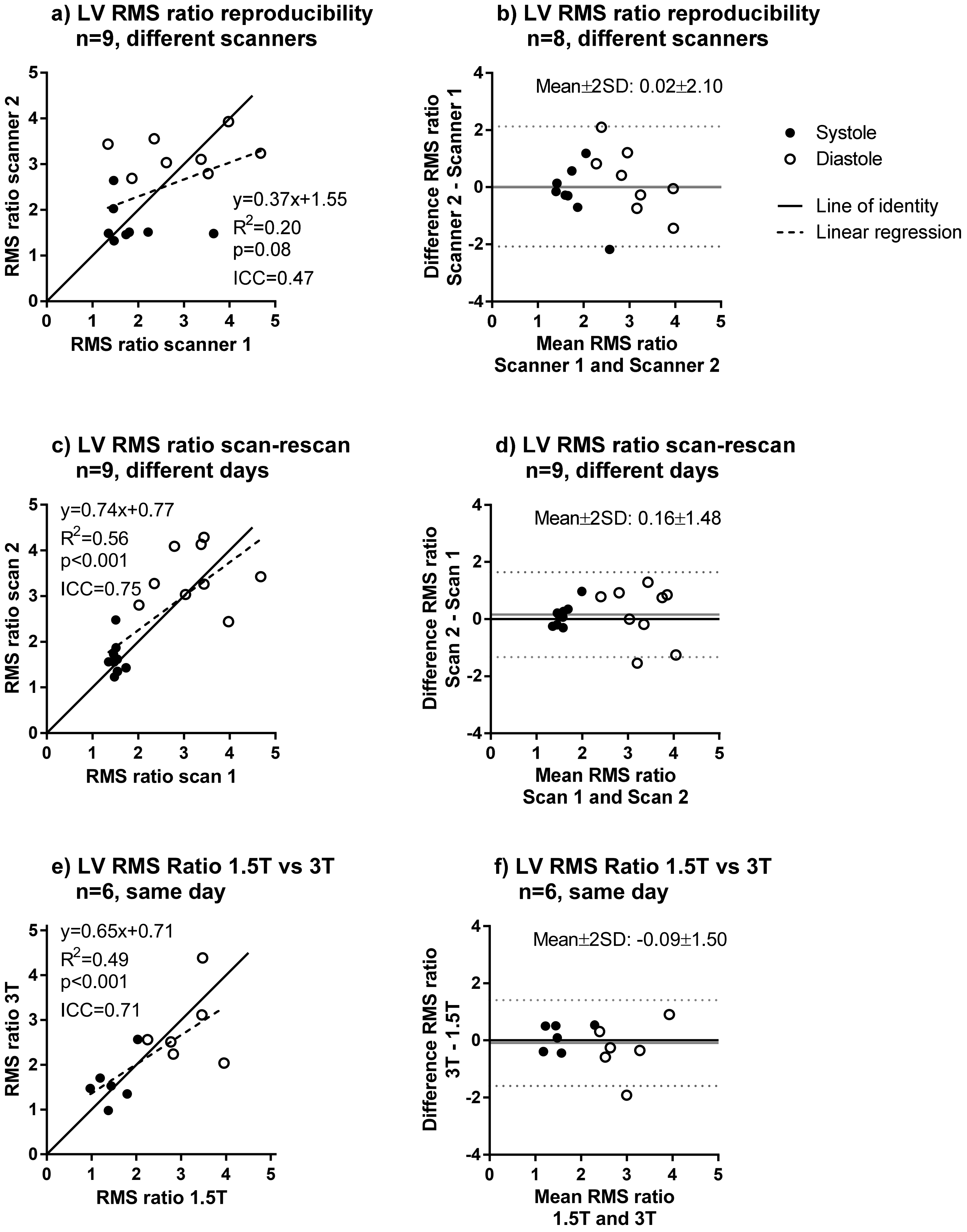
a) LV RMS ratio reproducibility
n=9, different scanners
RMS ratio scanner 2
RMS ratio scanner 1
y=0.37x+1.55
R2=0.20
p=0.08
ICC=0.47
b) LV RMS ratio reproducibility
n=8, different scanners
Mean±2SD: 0.02±2.10
Difference RMS ratio
Scanner 2 - Scanner 1
Mean RMS ratio
Scanner 1 and Scanner 2
Systole
Diastole
Line of identity
Linear regression
c) LV RMS ratio scan-rescan
n=9, different days
y=0.74x+0.77
R2=0.56
p<0.001
ICC=0.75
RMS ratio scan 2
RMS ratio scan 1
d) LV RMS ratio scan-rescan
n=9, different days
Mean±2SD: 0.16±1.48
Difference RMS ratio
Scan 2 - Scan 1
Mean RMS ratio
Scan 1 and Scan 2
e) LV RMS Ratio 1.5T vs 3T
n=6, same day
y=0.65x+0.71
R2=0.49
p<0.001
ICC=0.71
RMS ratio 3T
RMS ratio 1.5T
f) LV RMS Ratio 1.5T vs 3T
n=6, same day
Mean±2SD: -0.09±1.50
Difference RMS ratio
3T - 1.5T
Mean RMS ratio
1.5T and 3T

g) LV RMS Ratio Resp+ vs Resp-
n=17, same session

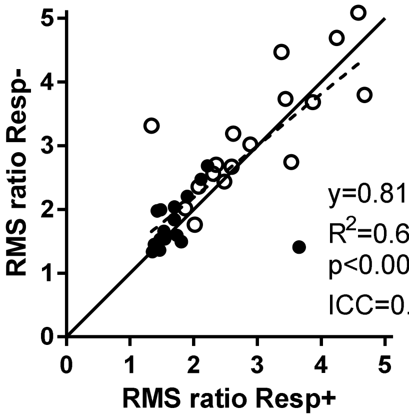

h) LV RMS ratio Resp+ vs Resp-
n=8, same session

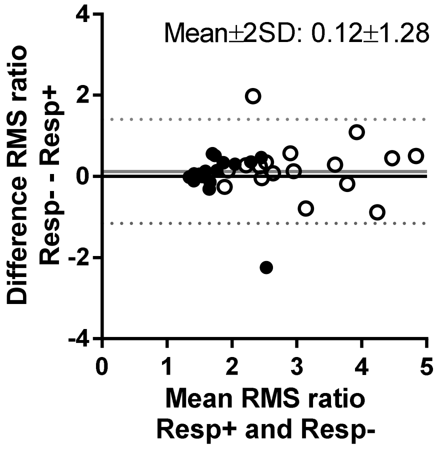

i) LV RMS Ratio manual vs auto
n=12, same dataset

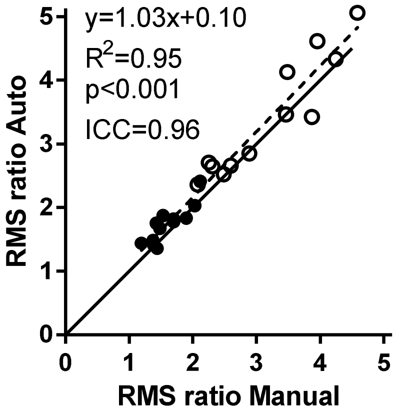

j) LV RMS Ratio manual vs auto
n=12, same dataset

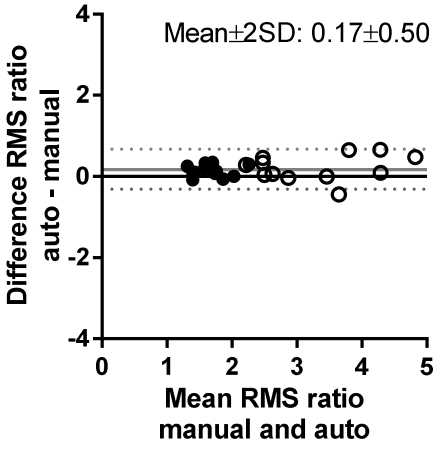

LV peak ratio

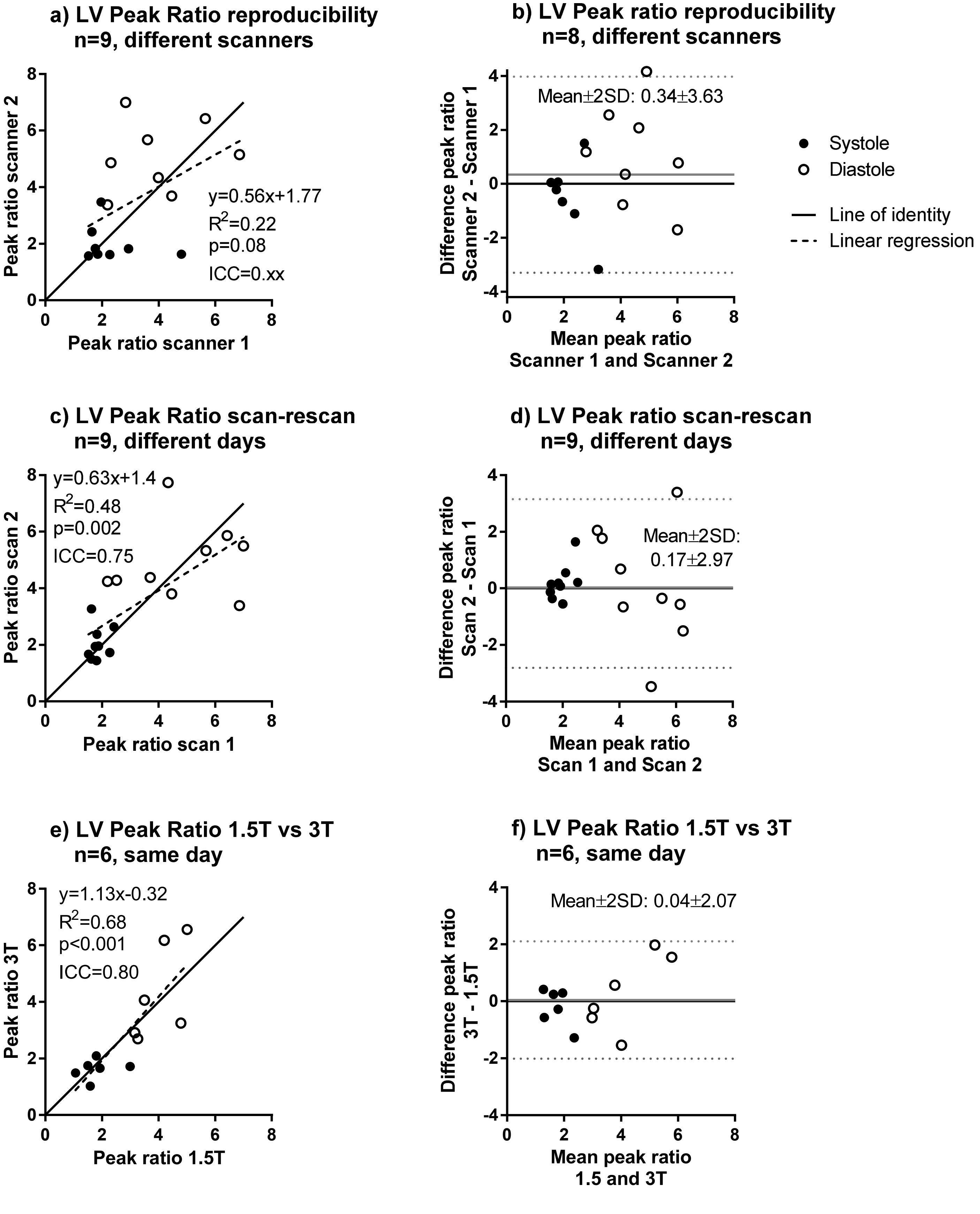

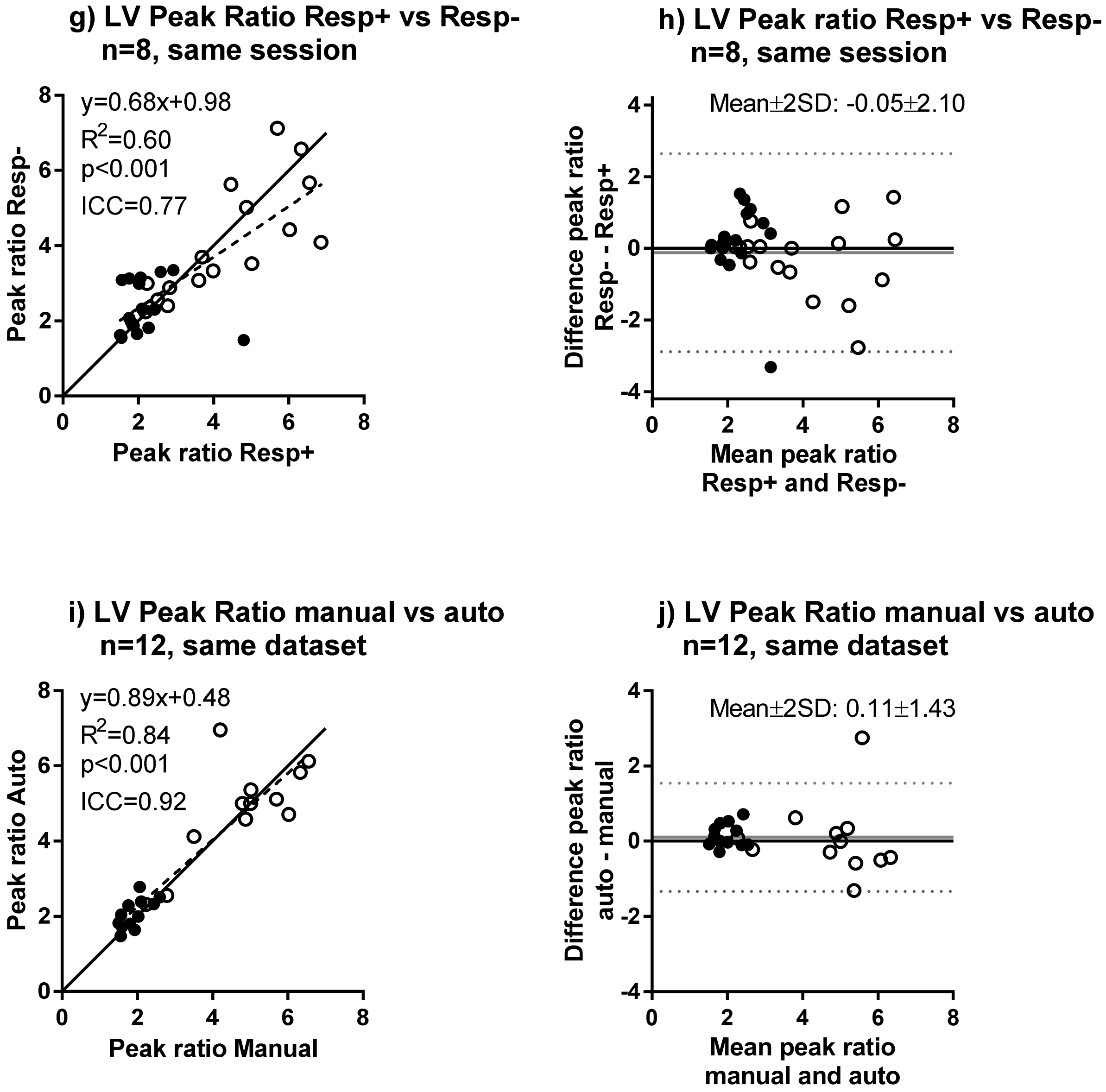
g) LV Peak Ratio Resp+ vs Resp-
n=8, same session
y=0.68x+0.98
R^2=0.60
p<0.001
ICC=0.77
Peak ratio Resp-
Peak ratio Resp+
0
2
4
6
8
h) LV Peak ratio Resp+ vs Resp-
n=8, same session
Mean±2SD: -0.05±2.10
Difference peak ratio
Resp- - Resp+
Mean peak ratio
Resp+ and Resp-
4
2
0
-2
-4
i) LV Peak Ratio manual vs auto
n=12, same dataset
y=0.89x+0.48
R^2=0.84
p<0.001
ICC=0.92
Peak ratio Auto
Peak ratio Manual
j) LV Peak Ratio manual vs auto
n=12, same dataset
Mean±2SD: 0.11±1.43
Difference peak ratio
auto - manual
Mean peak ratio
manual and auto
