# Supplementary material for: Hemodynamic forces in the left and right ventricles of the human heart using 4D flow magnetic resonance imaging: Phantom validation, reproducibility, sensitivity to respiratory gating and free analysis software
Source: PLoS One. 2018 Apr 5;13(4):e0195597. doi: 10.1371/journal.pone.0195597 (PMC5886587; doi:10.1371/journal.pone.0195597)

S4 Appendix: Graphical results for reproducibility of RV hemodynamic force measurements

Hemodynamic forces in the left and right ventricles of the human heart using 4D flow magnetic resonance imaging: reproducibility and sensitivity to respiratory gating, field strength and ventricle segmentation, with free analysis software

Johannes Töger[1], Per M Arvidsson[1], Jelena Bock[1], Mikael Kanski[1],

Gianni Pedrizzetti[2], Marcus Carlsson[1], Håkan Arheden[1], Einar Heiberg[1,3*]

[1]Lund University, Skane University Hospital, Department of Clinical Physiology, Lund, Sweden

[2]Department of Engineering and Architecture, University of Trieste, Trieste, Italy

[3]Department of Biomedical Engineering, Faculty of Engineering, Lund University, Lund, Sweden

PLOS One 2018, doi: 10.1371/journal.pone.0195597

*: Corresponding author: Einar Heiberg

Department of Clinical Physiology, Lund University Hospital, SE-22185 Lund, Sweden

einar.heiberg@med.lu.se

Phone: +46-46-171605, Fax: +46-46-151769

RV RMS forces

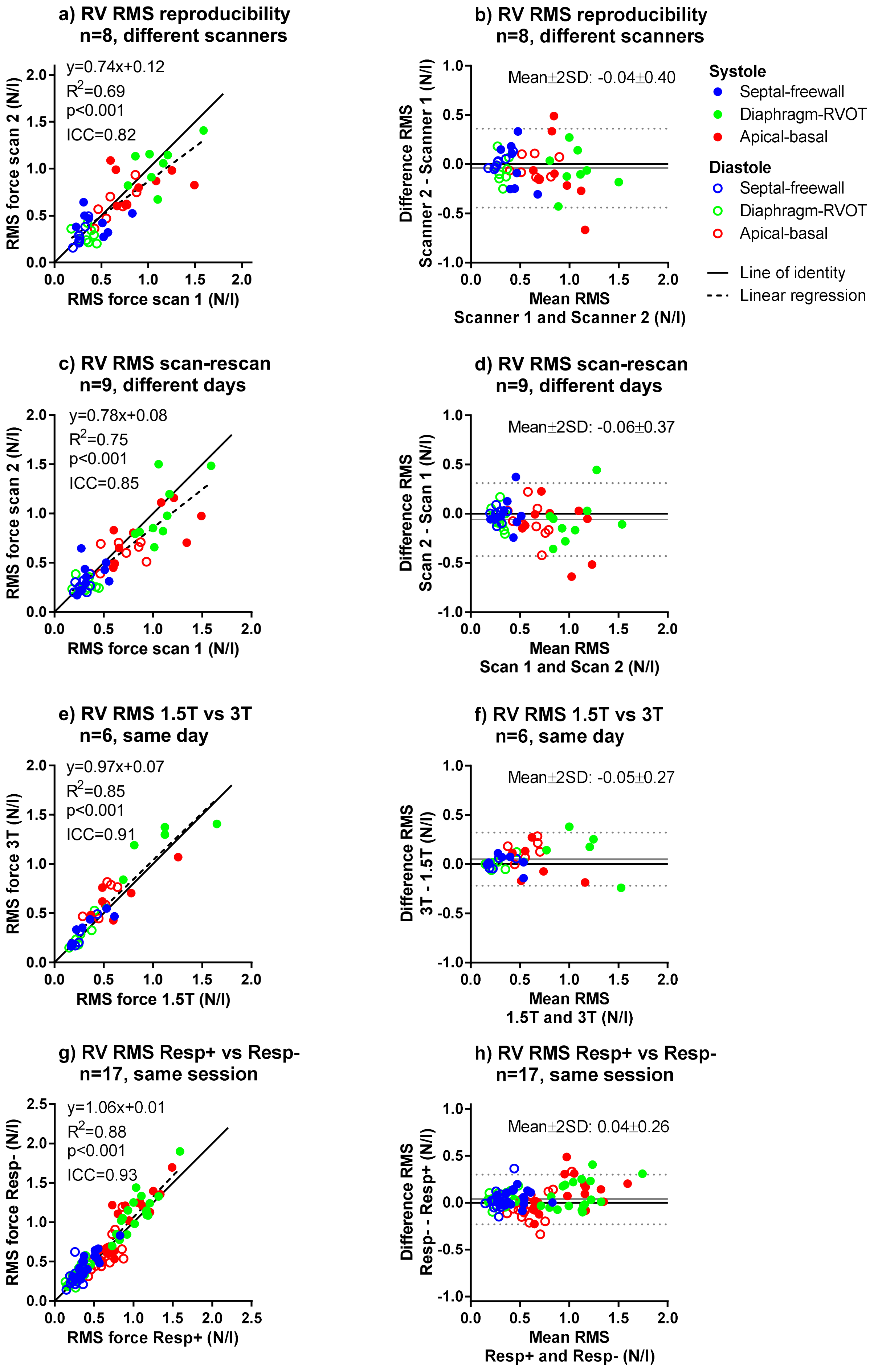

RV RMS ratio

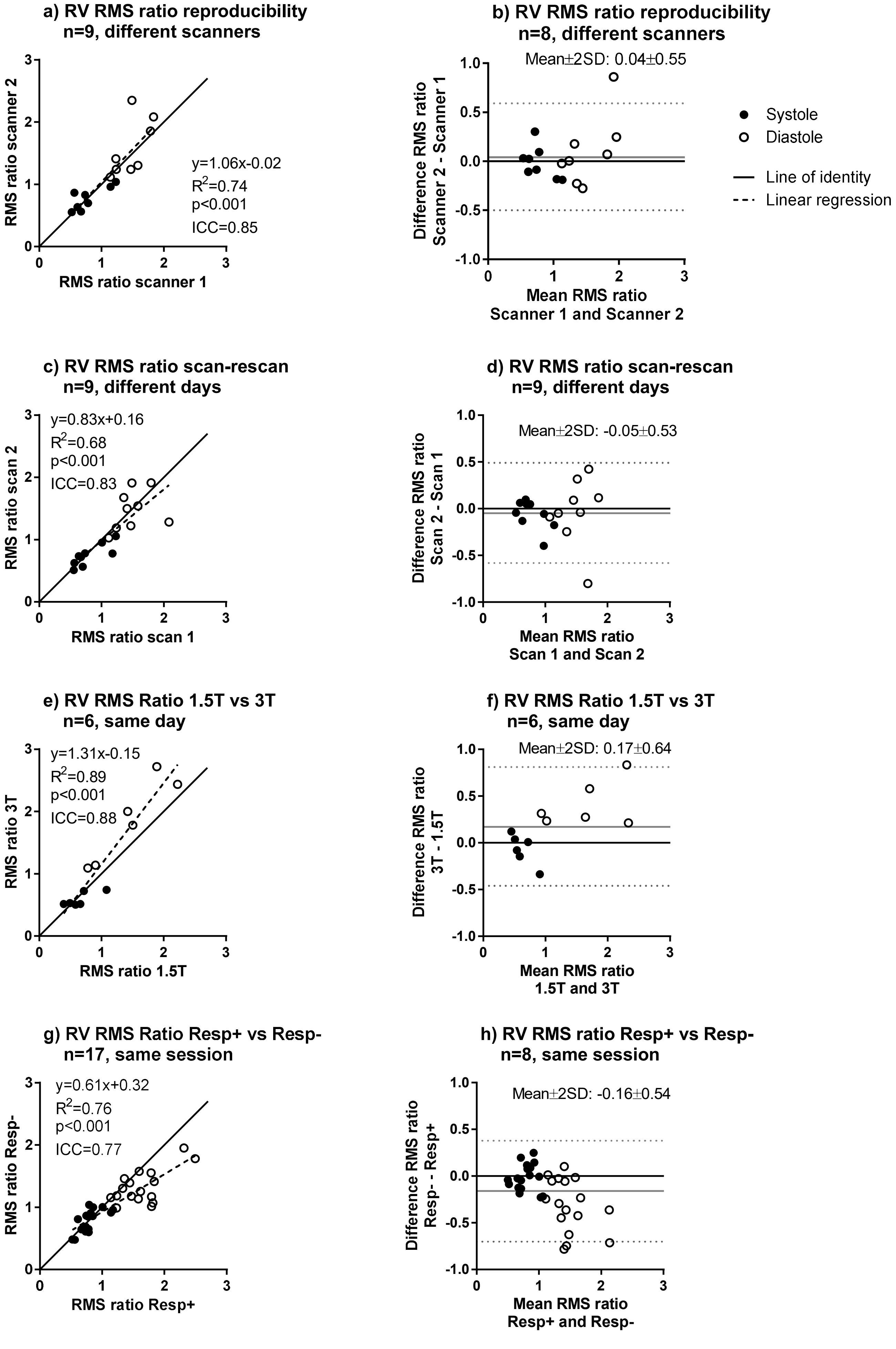

a) RV RMS ratio reproducibility
n=9, different scanners
RMS ratio scanner 2
RMS ratio scanner 1
y=1.06x-0.02
R2=0.74
p<0.001
ICC=0.85
b) RV RMS ratio reproducibility
n=8, different scanners
Mean±2SD: 0.04±0.55
Difference RMS ratio
Scanner 2 - Scanner 1
Mean RMS ratio
Scanner 1 and Scanner 2
Systole
Diastole
Line of identity
Linear regression
c) RV RMS ratio scan-rescan
n=9, different days
y=0.83x+0.16
R2=0.68
p<0.001
ICC=0.83
RMS ratio scan 2
RMS ratio scan 1
d) RV RMS ratio scan-rescan
n=9, different days
Mean±2SD: -0.05±0.53
Difference RMS ratio
Scan 2 - Scan 1
Mean RMS ratio
Scan 1 and Scan 2
e) RV RMS Ratio 1.5T vs 3T
n=6, same day
y=1.31x-0.15
R2=0.89
p<0.001
ICC=0.88
RMS ratio 3T
RMS ratio 1.5T
f) RV RMS Ratio 1.5T vs 3T
n=6, same day
Mean±2SD: 0.17±0.64
Difference RMS ratio
3T - 1.5T
Mean RMS ratio
1.5T and 3T
g) RV RMS Ratio Resp+ vs Resp-
n=17, same session
y=0.61x+0.32
R2=0.76
p<0.001
ICC=0.77
RMS ratio Resp-
RMS ratio Resp+
h) RV RMS ratio Resp+ vs Resp-
n=8, same session
Mean±2SD: -0.16±0.54
Difference RMS ratio
Resp- - Resp+
Mean RMS ratio
Resp+ and Resp-

RV peak forces

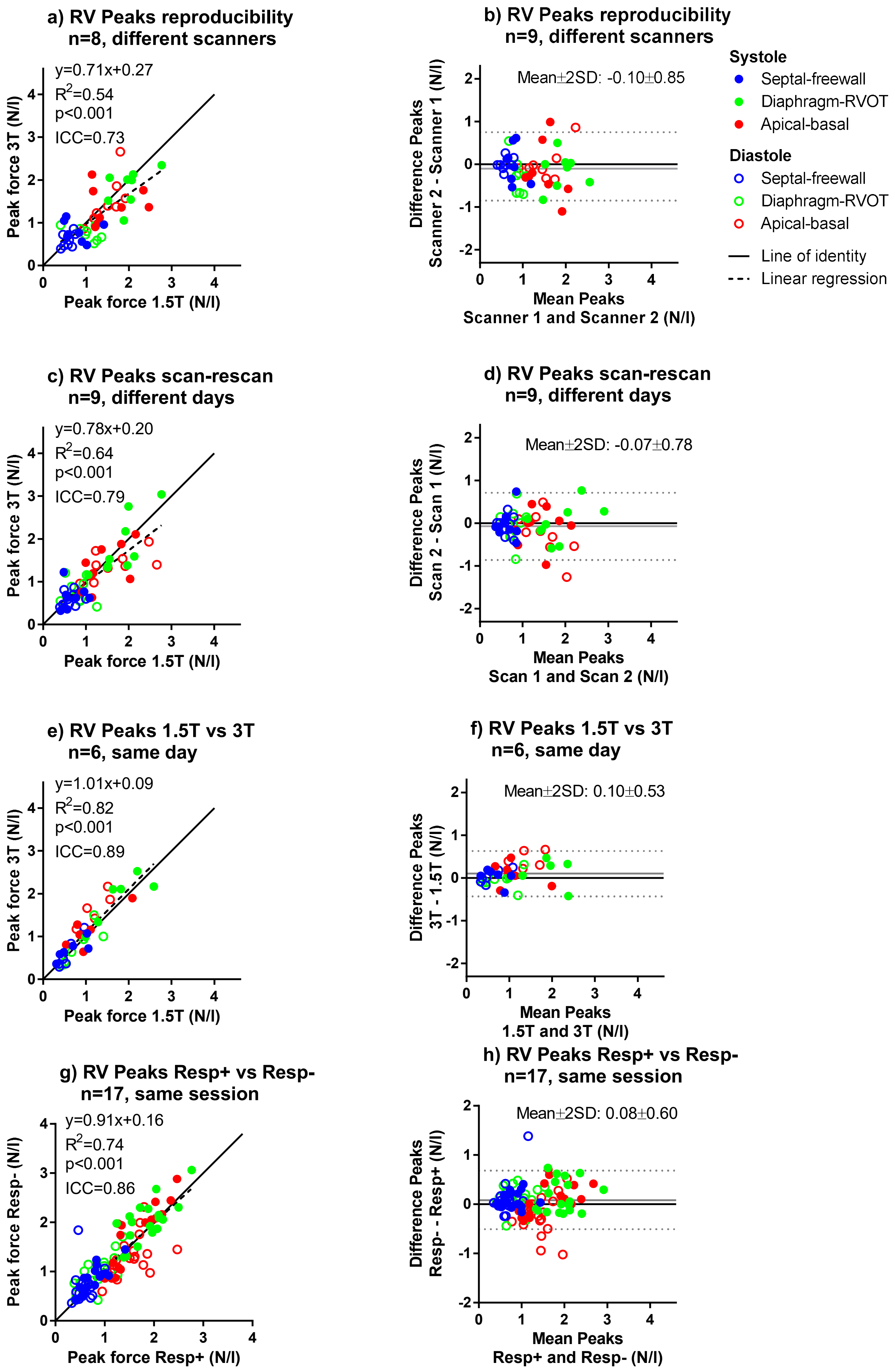

RV peak ratio

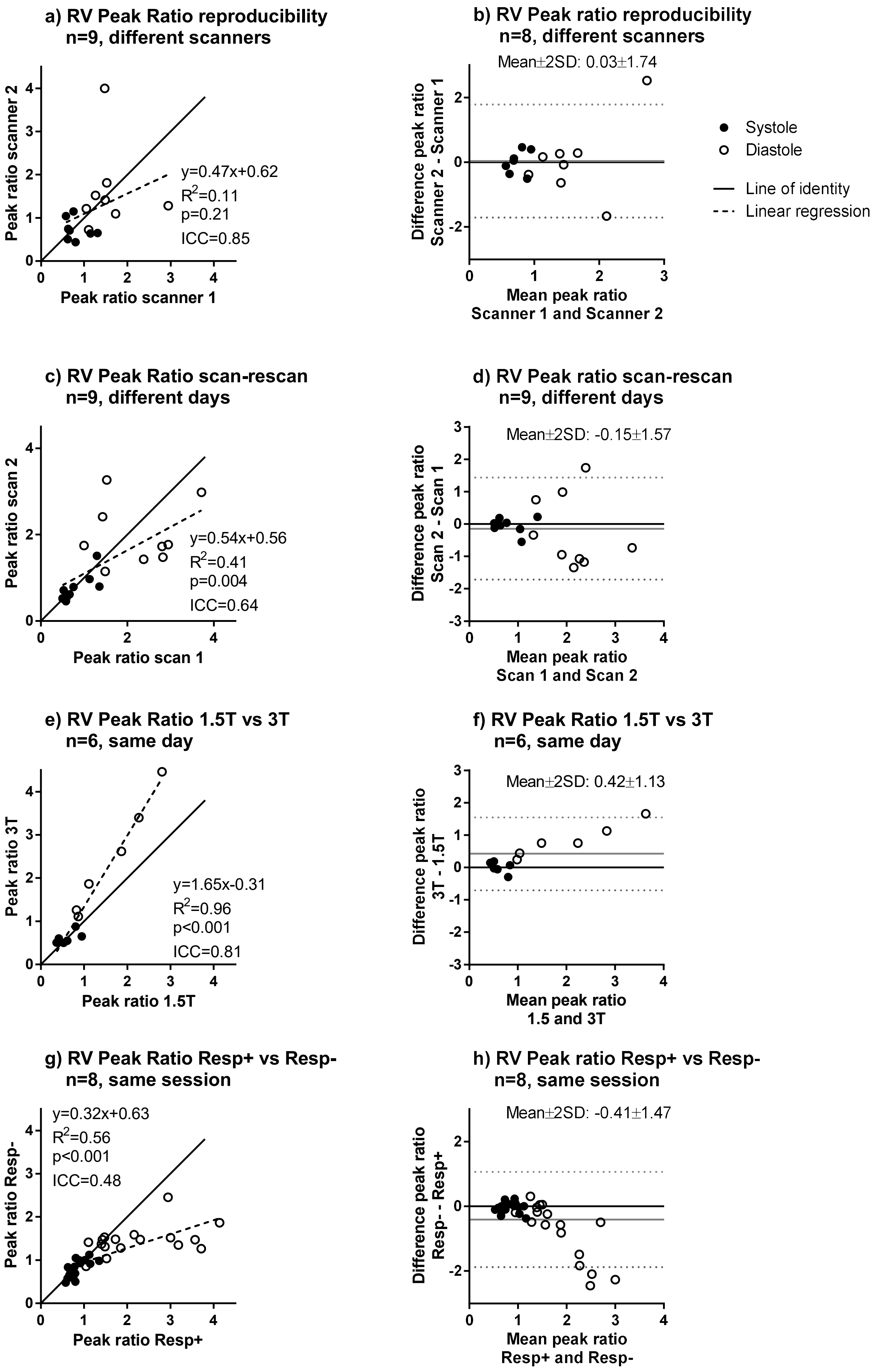

Supplement: S4 Appendix — Graphical results for reproducibility of right ventricular (RV) hemodynamic force measurements. (PDF) [file pone.0195597.s004.pdf]
